# Spatially explicit multi-threat assessment of food tree species in Burkina Faso: A fine-scale approach

**DOI:** 10.1371/journal.pone.0184457

**Published:** 2017-09-07

**Authors:** Hannes Gaisberger, Roeland Kindt, Judy Loo, Marco Schmidt, Fidèle Bognounou, Sié Sylvestre Da, Ousmane Boukary Diallo, Souleymane Ganaba, Assan Gnoumou, Djingdia Lompo, Anne Mette Lykke, Elisée Mbayngone, Blandine Marie Ivette Nacoulma, Moussa Ouedraogo, Oumarou Ouédraogo, Charles Parkouda, Stefan Porembski, Patrice Savadogo, Adjima Thiombiano, Guibien Zerbo, Barbara Vinceti

**Affiliations:** 1 Bioversity International, Via dei Tre Denari 472/a, Maccarese (Rome), Italy; 2 World Agroforestry Centre (ICRAF), Nairobi, Kenya; 3 Senckenberg Biodiversity and Climate Research Centre, Data and Modelling Centre, Senckenberganlage 25, Frankfurt, Germany; 4 University of Quebec, 2600 Boulevard Laurier, Ville de Québec, QC, Canada; 5 West African Science Service Center on Climate Change and Adapted Land Use (WASCAL), Blvd Mouammar Kadhafi, Ouagadougou, Burkina Faso; 6 Environmental and Agricultural Research Institute, INERA/CNRST, Ouagadougou, Burkina Faso; 7 University Aube Nouvelle, Ouagadougou, Burkina Faso; 8 National Forest Seed Centre, CNSF, Ouagadougou, Burkina Faso; 9 Aarhus University, Vejlsøvej 25, Silkeborg, Denmark; 10 University of N'Djaména, Avenue Mobutu, N'Djamena, Chad; 11 Laboratory of Botany and Plant Ecology, University of Ouagadougou, Ouagadougou, Burkina Faso; 12 Department of Food Technology, IRSAT/CNRST, DTA, Ouagadougou, Burkina Faso; 13 University of Rostock, Rostock, Germany; 14 World Agroforestry Centre & International Crop Research Institute for the Semi-Arid Tropics (ICRAF-ICRISAT), Niamey, Niger; Pacific Northwest National Laboratory, UNITED STATES

## Abstract

Over the last decades agroforestry parklands in Burkina Faso have come under increasing demographic as well as climatic pressures, which are threatening indigenous tree species that contribute substantially to income generation and nutrition in rural households. Analyzing the threats as well as the species vulnerability to them is fundamental for priority setting in conservation planning. Guided by literature and local experts we selected 16 important food tree species (*Acacia macrostachya*, *Acacia senegal*, *Adansonia digitata*, *Annona senegalensis*, *Balanites aegyptiaca*, *Bombax costatum*, *Boscia senegalensis*, *Detarium microcarpum*, *Lannea microcarpa*, *Parkia biglobosa*, *Sclerocarya birrea*, *Strychnos spinosa*, *Tamarindus indica*, *Vitellaria paradoxa*, *Ximenia americana*, *Ziziphus mauritiana*) and six key threats to them (overexploitation, overgrazing, fire, cotton production, mining and climate change). We developed a species-specific and spatially explicit approach combining freely accessible datasets, species distribution models (SDMs), climate models and expert survey results to predict, at fine scale, where these threats are likely to have the greatest impact. We find that all species face serious threats throughout much of their distribution in Burkina Faso and that climate change is predicted to be the most prevalent threat in the long term, whereas overexploitation and cotton production are the most important short-term threats. Tree populations growing in areas designated as ‘highly threatened’ due to climate change should be used as seed sources for *ex situ* conservation and planting in areas where future climate is predicting suitable habitats. Assisted regeneration is suggested for populations in areas where suitable habitat under future climate conditions coincides with high threat levels due to short-term threats. In the case of *Vitellaria paradoxa*, we suggest collecting seed along the northern margins of its distribution and considering assisted regeneration in the central part where the current threat level is high due to overexploitation. In the same way, population-specific recommendations can be derived from the individual and combined threat maps of the other 15 food tree species. The approach can be easily transferred to other countries and can be used to analyze general and species specific threats at finer and more local as well as at broader (continental) scales in order to plan more selective and efficient conservation actions in time. The concept can be applied anywhere as long as appropriate spatial data are available as well as knowledgeable experts.

## Introduction

Burkina Faso is among the poorest countries in the world, with a very low Human Development Index (HDI), holding position 183 out of 188 countries [1]. About 90% of its 17 million inhabitants rely on subsistence agriculture and livestock farming for their livelihoods. As in many other countries in Western Africa, most rural communities greatly depend on goods provided by trees and woodland environments; these include food, timber, fuelwood, medicine and animal fodder [2,3]. The country can be divided into three eco-climatic zones, based on annual average rainfall distribution: i) the Sahelian zone in the northern part of the country, with annual rainfall below 600 mm spread over a period of three to four months and vegetation characterized by dry savanna with sparse tree cover; ii) a transitional Sudano-Sahelian zone in the central region, with a total rainfall of 600–900 mm distributed over four to five months; and iii) the Sudanian zone in the southern part, with annual average rainfall of more than 900 millimeters spread over five to six months [2,4].

Agroforestry parklands are among the most widespread traditional land use systems in Burkina Faso, as in many other parts of sub-Saharan Africa, where scattered individual mature trees occur on cultivated fields [5]. Due to the value and variety of their products, trees in parkland systems are retained by farmers when woodland and old fallows are converted into cropland [6,7]. In this way, low annual crop yields are offset by the availability of non-timber forest products, which contribute substantially to income generation, nutrition and food security [8–14].

Natural resources are overexploited in some parts of the country, due to the increasing demographic pressure and human migration associated with overgrazing and environmental changes [15,16]. Conflicting land use activities include uncontrolled bush burning, extensive cattle grazing, and deforestation to clear land for agriculture [17]. Additionally, the century or millennia-long preference for edible-fruit-yielding taxa from the wetter Sudanian and Guinean vegetation zones over Sahelian species in parkland systems appears to have stranded species that have anthropogenic-driven distribution beyond their rainfall tolerance limits, after the sharp drop in precipitation since the 1960s [18]. Due to these factors, important and valued indigenous food tree species (e.g., *Vitellaria paradoxa* C. F. Gaertn., *Tamarindus indica* L., *Detarium microcarpum* Guill. & Perr., *Parkia biglobosa* (Jacq.) G. Don are increasingly vulnerable to various drivers of change, such as harvesting for fuelwood or charcoal production, removal of trees in intensive cotton agriculture and increasing frequency and intensity of droughts. Climate change, in particular, is likely to intensify natural tree regeneration problems and progressively modify the distribution of suitable habitats for several tree species, in parts of their range, as the result of shifts in the main vegetation and eco-climatic zones [18–20].

Understanding the combined effects of different threats on the distribution of important indigenous food tree species in Burkina Faso is necessary for priority setting in conservation planning. Previous attempts to rank threats to forest cover included examining potential changes in biodiversity at a larger regional or ecoregional scale [21–23] and combining these layers with IUCN red listing categories [24]. However, no method has been developed so far to generate species-specific threat layers.

In this paper we present a framework to develop a species-specific threat assessment designed to predict, at the population level, where multiple threats (e.g. overexploitation, overgrazing, projected impact of climate change) are likely to have a negative impact on the availability of suitable habitat in the present and near future. Thus, the main objectives of this study were to:

1. Develop a species-specific and spatially explicit threat model based on freely accessible (global and local) datasets, calibrated using local expert knowledge;
2. Identify areas where important food tree species in Burkina Faso are highly threatened using spatial analysis for each species and threat factor individually and in combination;
3. Recommend conservation actions for priority tree populations.

## Materials and methods

### Selection of important food tree species and potential threats

The focus of this analysis is on the main present and future threats to important food tree species in Burkina Faso. In this context, threats can be defined as proximate activities or processes that have caused, are causing, or may cause the destruction, degradation, and/or impairment of biodiversity targets [25]. Potential threats were identified from the literature, through consultation of local experts and by means of a case study on farmers’ perception. Subsequently we chose the most appropriate datasets to describe the spatial patterns of threat throughout the country (Table 1). Climate change and its impacts were projected until 2055.

**Table 1:** Threat layers and respective data sources.

Key threats	Indicators	Spatial layers	Impact at population level
Overexploitation *▪	Population density, human land use and infrastructure	WCS and CIESIN/Columbia University. 2005. Last of the Wild Project, Version 2, 2005. Global Human Footprint ●	Fragments populations, reduces tree density
Overgrazing *▪	Cattle, goat and sheep density per area	Gridded Livestock of the World v2.0, 2014, FAO and ILRI ●	Inhibits natural regeneration
Fire *▪	Fire frequency per unit area	NASA Fire Information for Resource Management System (FIRMS), 2012. MODIS Active Fire Detections 2007–2012 ●	Reduces tree density, inhibits natural regeneration
Cotton production **▪	Cotton growing area as percentage of land area	Ministry Report 2010 map and regional cotton production statistics ●	Eliminates natural populations
Mining **▪	Presence of mining sites, occurrences and prospects	Mineral Resources Data System (MRDS) of the U.S. Geological Survey 2005 + Ministry Report 2010 map ●●	Eliminates natural populations; opens roads
Climate change *▪▪	Predicted absence, presence or presence under novel regional climatic conditions of suitable habitat	Bioclimatic dataset under future conditions: Downscaled GCMs from CMIP5 for 2055, RCP 4.5 and 8.5 scenarios ●●	Reduces flowering and fruit set, damages healthy individuals, promotes invasive species

* species-specific threat.

** generic threat.

▪ short-term threat.

▪▪ long-term threat.

● spatial layer with quantitative data.

●● spatial layer with qualitative data.

A list of indigenous food tree species that are important in Burkina Faso was prepared based on a literature review and expert consultations [26–31]. From this initial list, 16 food tree species were selected (Table 2) based on the availability of occurrence data for species distribution modeling and the Plant Resources of Tropical Africa (PROTA) star-ratings (https://www.prota4u.org/database/starratings.asp) for vegetable, fruit and carbohydrate-starch use importance. The species selected had a minimum of 30 unique observations and a minimum rating of 2 (star-ratings range from 1 to 5) for at least one of the categories.

**Table 2:** Expert survey results on SDMs based on the consensus approach.

Species	Distribution Model	Weighted Score	Number of Experts	Average Concordance Value
*Acacia macrostachya* Rchb. ex DC.	1	3.16	14	0.30
	2	1.93		
	**3**	**3.51**		
	4	2.79		
*Acacia senegal* (L.) Willd.	**1**	**3.99**	9	0.58
	2	2.43		
	3	2.15		
	4	1.83		
*Adansonia digitata* L.	1	2.48	14	0.74
	2	2.51		
	3	3.5		
	**4**	**4.3**		
*Annona senegalensis* Pers.	1	2.37	13	0.50
	**2**	**3.67**		
	3	2.29		
	4	3.55		
*Balanites aegyptiaca* (L.) Delile	1	1.65	13	0.59
	2	1.91		
	3	1.85		
	**4**	**3.93**		
*Bombax costatum* Pellegr. & Vuill.	1	2.67	14	0.77
	2	1.51		
	3	2.88		
	**4**	**3.92**		
*Boscia senegalensis* (Pers.) Lam.	**1**	**4**	13	0.58
	2	2.49		
	3	2.71		
	4	1.57		
*Detarium microcarpum* Guill. & Perr.	1	2.23	15	0.38
	**2**	**4.1**		
	3	1.91		
	4	2.43		
*Lannea microcarpa* Engl. & K. Krause	**1**	**3.74**	14	0.63
	2	1.21		
	3	2.48		
	4	3.35		
*Parkia biglobosa* (Jacq.) G. Don	1	2.8	14	0.57
	**2**	**4.02**		
	3	2.37		
	4	2.85		
*Sclerocarya birrea* (A. Rich.) Hochst.	1	1.81	13	0.41
	2	1.81		
	**3**	**3.76**		
	4	1.91		
*Strychnos spinosa* Lam.	1	2.58	13	0.43
	**2**	**4.15**		
	3	2.59		
	4	3.15		
*Tamarindus indica* L.	1	2.74	13	0.42
	**2**	**4.29**		
	3	2.51		
	4	2.87		
*Vitellaria paradoxa* C. F. Gaertn.	1	2.69	15	0.63
	**2**	**4.4**		
	3	2.34		
	4	2.74		
*Ximenia americana* L.	1	2.55	13	0.47
	2	2.21		
	3	2.23		
	**4**	**3.84**		
*Ziziphus mauritiana* Lam.	1	2.06	12	0.64
	**2**	**3.9**		
	3	2.3		
	4	2.9		

The most appropriate species distribution model (SDM), marked in bold, is selected based on the highest weighted score. Model 1: annual variables, 90% threshold; Model 2: annual variables, ‘ensemble.min’ threshold; Model 3: bioclimatic subset of variables, 90% threshold and Model 4: bioclimatic subset of variables, ‘ensemble.min’ threshold. Weighted score values can vary between 1 and 5. In addition, the table shows the number of valid expert responses (number of experts) and the average expert concordance (average concordance value) per species.

### Modeled potential distribution

Tree distribution data were compiled from a Burkina Faso tree species distribution map prepared by Terrible [32](S1 Dataset); collection data from the Herbarium Senckenbergianum (FR) and the Aarhus University (AAU) herbarium from the West African Vegetation Database [33] (S1 Dataset); georeferenced photo records of African Plants [34] (S1 Dataset); observation records from a transect study of useful tree species in western Burkina Faso [35] (S1 Dataset); and point locations obtained from the Global Biodiversity Information Facility [36], available within the range of 18 degrees west to 16 degrees east in longitude and latitudes of 4 to 28 degrees north.

Suitability models were calibrated with BiodiversityR [37] using an ensemble suitability modeling approach that fitted an ensemble model based on the 10 submodels (different algorithms that predict suitability such as random forests, boosted regression trees or artificial networks; the ensemble suitability is calculated as weighted average of these suitabilities) with highest average AUC (Area Under the Receiver-operator curve). The average AUC of 12 candidate submodels was calculated from testing data after splitting the presence and absence locations in 4 random subsets [38,39] and was used as weight in the calculation of the ensemble suitability. In one series of model calibrations, explanatory variables were bioclimatic ones obtained from AFRICLIM [40] with a resolution of 2.5 arc-minutes, calibrated from the WorldClim 1.4 as baseline current climate [41]. The variables had a maximum Variance Inflation Factor (VIF) of 20, selected by a stepwise process whereby the variables with highest VIF were removed in each step, resulting in retaining: (i) BIO1 (annual mean temperature, VIF 3.78); (ii) BIO3 (isothermality, VIF 15.04); (iii) BIO5 (maximum temperature of the warmest month, VIF 13.31); (iv) BIO13 (precipitation of the wettest month, VIF 4.46); (v) BIO14 (precipitation of the driest month, VIF 1.77); (vi) LLDS (length of the longest dry season, VIF 13.31); and (vii) PET (potential evapotranspiration, VIF 6.80). In another series of model calibrations, we only used (i) BIO1 (annual mean temperature) and BIO12 (annual precipitation). Species suitability values were obtained as averages from repeating the model calibration procedure (including random selection of 2000 background locations, splitting presence and absence locations in random subsets and calibrating submodels) five times for each subset of explanatory variables.

A series of four potential distribution maps was produced for each species, using the different combinations of two different subsets of explanatory variables (one based on the VIF and one on annual mean temperature and annual precipitation) and two threshold levels to differentiate between species absence-presence (BiodiversityR function *ensemble*.*test* with option ‘minimum’ and ‘true positive rate of 0.90’ (two thresholds used in a previous study [42]). The methodology of different combinations of explanatory variables and threshold values was aimed at producing a series of current distribution maps that significantly differed in the areas mapped as suitable for each species, a requirement for expert comparisons of candidate suitability maps. By juxtaposing and visually comparing the different distribution maps for each species, we confirmed that our methods produced four clear alternatives of current distribution of each species that could be subjected to expert evaluation.

### Expert evaluation of species distribution

Seventeen experts with botanical expertise and knowledge of Burkina Faso were involved in assessing the representativeness of the current spatial distribution models elaborated for the 16 selected tree species by means of an online survey (S1 Appendix). Most of them had detailed knowledge of all the selected food tree species and some focused on particular species within their expertise. For each of the 16 tree species, the four SDMs were ranked by each expert independently on a five-point scale from 1 (not valid) to 5 (excellent), based on how well the model represented the species distribution; furthermore, the experts individually attributed to each tree species a sensitivity value to the different threats selected (S1 Appendix).

A consensus theory approach was applied [42] to formalize the experts’ feedback. Through the consensus approach, the degree of concordance among individual experts’ feedback can be estimated. For each expert the degree of concordance with the other experts was calculated as pairwise Spearman correlation coefficient. Then a maximum-likelihood factor analysis was carried out on the correlation coefficient matrices. The amount of variance explained in the first factor indicated the rate of consensus between experts on best model selection. The degree of concordance, which can vary between 0 and 1, was used to weight each informant’s response. This response contributed to the definition of a final score for each model (Table 2) which was used to identify the most appropriate distribution map for each tree species.

The average concordance rates were also used to indicate the consistency of the expert group to accurately estimate the best distribution model. For each species, the SDM that obtained the highest weighted score was used as the basis for further analyses.

### Data sources for main threats

The study covers all of Burkina Faso with a spatial resolution of 2.5 arc-minutes (about 4.5 km at the equator), based on the resolution of the bioclimatic variables used for the suitability models. Threat layers with different resolutions were resampled in ArcGIS 10.1 using bilinear interpolation. Furthermore, a square-root transformation was applied to the layers ‘overgrazing’ and ‘fire’ due to the heavy skewness in the frequency distributions. To facilitate calculations, the raster layers with quantitative data (overexploitation, overgrazing, fire and cotton production) were normalized on a pixel-by-pixel basis to obtain values between 0 and 1.

#### Overexploitation

To assess the threat potential due to overexploitation, which includes harvesting for food, timber, fuelwood, and animal fodder, we used the Global Human Footprint dataset [43]. It is the result of various global layers representing anthropogenic factors presumed to exert an influence on the ecosystem: human population density, type of land use system, infrastructure distribution (built-up areas, nighttime lights, land use/land cover) and accessibility (coastlines, roads, railroads, navigable rivers). The values range from 0 to 100 per pixel and indicate the intensity of human influence on an area.

#### Overgrazing

The Gridded Livestock of the World v2.0 [44] was used to assess the threat associated with the pressure of livestock through grazing, likely affecting tree regeneration. The modeled livestock density combines statistics at province level within Burkina Faso with remote sensing data on climate, environment, demography, land cover and terrain. The tropical livestock units (TLUs), a common measure used to standardize livestock numbers by summing cattle, goats and sheep densities, was calculated using the function ‘Raster calculator’ in ArcGIS 10.1. A TLU is the grazing equivalent of an animal ruminant of 250 kilograms live weight, and the TLU conversion factors used are: cattle 0.7, goats and sheep 0.1 [45]. The TLUs per pixel ranged from 0 to 170.

#### Fire

To assess the spatial pattern of fires caused by natural events or human activities, we utilized MODIS FIRMS data [46] for Burkina Faso between 2007 and 2012. Each active fire detection represents the center of a pixel approximately 1 km^2^ in size flagged as containing one or more fires. Fire events were included only if the detection confidence exceeded 30%, and a selection of 113,636 single occurrences in Burkina Faso was retained to calculate fire frequency per km^2^. We distinguished early fires, taking place at the beginning of the dry season, from late fires, usually more intense and occurring during the peak of the dry season (January to March) when the vegetation is completely dry and the fuel load is higher. Double weight was assigned to late fires because mortality rates of trees can be twice as high in late-burned plots as in early-burned plots [47]. Mean annual fire frequency per km² was determined with the functions ‘Cell statistics’ and ‘Raster calculator’ in ArcGIS 10.1, by averaging fire frequencies per year from 2007 to 2012. The mean annual fire frequencies per pixel ranged from 0 to 1.35.

#### Cotton production

Burkina Faso is the number one cotton producer in West Africa and production has increased rapidly over the past two decades. Currently, cotton farms account for more than 6% of the country’s agricultural land [48,49]. To assess the threat posed to the tree cover due to conversion of woodland or agroforestry parklands into intensified cotton production, we georeferenced the most recent national map showing cotton production areas [50] and combined it with production statistics at province level [51]. A cotton production intensity map was created by dividing the production value within each province equally among the pixels on which cotton is produced. The production area is presented as a percentage of the total land area, with values ranging from 0 to 16.4 per pixel.

#### Mining

Whereas cotton accounted for 80% of exports in Burkina Faso and gold production was non-existent a decade ago, gold now accounts for almost 80% of exports [49]. Burkina Faso is ranked fourth in Africa for gold production, and it has the third highest exploration activity in the continent; production is expected to continue its rapid growth. The Mineral Resource Data System [52], representing sites where mineral commodities are known or likely to be present, was used to assess the threat of mining activities on the selected food tree species. The influence of mining activities includes habitat destruction in the mining sites and habitat degradation in the surrounding areas. Active mining sites (presently exploited or with prospects of future exploitation) were selected. Additional sites where gold, manganese and phosphate mines are located were included by georeferencing the most recent national map of mining sites [50]. Mining points were converted into single raster pixels with the function “point to raster” in ArcGIS 10.1.

#### Climate change

Climate projections for the middle of the 21^st^ century (2041–2070) were obtained from AFRICLIM [40], consisting of downscaled data from General Circulation Model (GCM) at a spatial resolution of 2.5 arc-minutes for intermediate and high greenhouse gas concentration pathways. The average ensembles for RCP 4.5 (raster layers corresponding to the bioclimatic conditions for intermediate Representative Concentration Pathway 4.5 W m^-2^) and RCP 8.5 were used to project future suitability with the most appropriate distribution map for each of the 16 target tree species.

We distinguished ‘novel regional conditions’ in our definition of threat levels associated with climate change because we noticed that calibrated models tended to predict species presence in some of the areas having novel climatic conditions, i.e. that the models extrapolated in environmental space outside the range of conditions occurring in Burkina Faso and in the larger region of West Africa to which the present species might not be adapted.

Climate change threat maps were created to depict: (i) very high threat: predicted absence of suitable habitat for the species in question for either RCP 4.5 or RCP 8.5 scenarios; (ii) high threat: predicted presence of suitable habitat for both RCP 4.5 and RCP 8.5 scenarios in novel regional climate conditions (novel conditions were mapped with BiodiversityR function *ensemble*.*novel–*these correspond to areas that are outside the minimum-maximum range of current conditions for at least one bioclimatic variable); (iii) medium threat: predicted presence of suitable habitat for RCP 4.5 scenario in novel regional climate conditions; (iv) low threat: predicted presence of suitable habitat for RCP 4.5; and (v) no threat: predicted presence of suitable habitat for RCP 4.5 and RCP 8.5 scenarios.

### Threat magnitude rating

To estimate the magnitude of a threat at a given location, for each tree species selected, we adopted the approach recommended by TNC [53] and combined geographic scope (area) and severity (intensity) of each individual threat. The geographic scope is defined by the proportion of the modeled species distribution that is affected by the threat and the severity by the intensity of the threat variable in each pixel (unit of measurement converted to values between 0 and 1). Threat intensity values were ranked based on a five-point rating scale (Table 3), as five classes were expected to provide sufficient spread without creating false precision (adapted from [54]). We assumed that the geographic scope and severity of a threat are maintained over time. Thus, the estimated threat magnitude indicates the combination of predicted and actual threat impact [54]. The threat magnitude rating deliberately contains non-linear cut-off values (Table 3) to reflect the non-linear nature of the relationship between intensity and potential impact, for most of the threats examined. Field experiments revealed that a threat often begins to take effect at a certain threshold, and the impact becomes severe with an increasing intensity until reaching a plateau [22,23,54].

**Table 3:** Five-point rating scale to define the potential threat magnitude.

Threat magnitude	Definition
Very high	The threat is likely to destroy or eliminate the species, or reduce its population by 71–100%
High	The threat is likely to seriously degrade/reduce the species or reduce its population by 31–70%
Medium	The threat is likely to moderately degrade/reduce the species or reduce its population by 11–30%
Low	The threat is likely to only slightly degrade/reduce the species or reduce its population by 1–10%
No threat	The threat is likely to not degrade/reduce the species or reduce its population by less than 1%

The definition of the threat magnitude classes and its non-linear cut-off values (adapted from [54]).

The dataset used to map ‘Cotton production’ had an insufficient spatial resolution to identify peaks of production intensity; thus, the maximum threat level was set to ‘High’.

The threat potential of each mining site was represented by a single 20.25 km^2^ pixel to which we assigned the threat level ‘Very high’. The coarse spatial resolution includes mining sites and degraded surrounding areas and therefore no further extrapolation was required. Cotton production and mining usually result in complete clearance of tree cover, therefore they do not pose species-specific threats. Thus, the threat intensity values of these two variables together with the modeled climate change variable were directly classified according to the authors’ best judgement supported by the definitions of the threat magnitude classes definition in Table 3; for the other variables (overexploitation, overgrazing and fire) the attribution of a threat magnitude was further guided by an expert validation, aimed at defining species specific vulnerability to different threats.

### Expert evaluation of threat sensitivity

To our knowledge no systematic methodology has been developed yet that incorporates expert opinion to determine species-specific threat sensitivity to drivers of change and population decline. In this paper, we propose obtaining expert feedback by means of an online survey (S1 Appendix), to systematically calibrate the spatial distribution of threat magnitude for each individual species. Expert-based methods allow the involvement of stakeholders in priority setting for conservation and have been used as a practical tool to take advantage of people’s experience and expertise in decision-making for natural resource management [55] and validation of species distribution models [42]. Cultural consensus theory provides a framework to formalize expert feedback for scientific analysis by estimating the competence of experts and weighting their feedback accordingly in final threat sensitivity scores [56].

The seventeen experts who assessed the representativeness of the spatial distribution models for the 16 selected tree species also individually attributed to each tree species a sensitivity value to the different threats selected (S1 Appendix). The experts’ feedback was formalized using the same consensus theory approach [42] as described above to evaluate the species distribution modeling, and the concordance rate, which varies between 0 and 1, was used to weight each expert’s response. The responses contributed to a species-specific sensitivity value (Table 4) to different threats, which was used to identify the sensitivity for each tree species to various threats.

**Table 4:** Expert survey results on threat sensitivity based on the consensus approach.

Species	Threat	Weighted Score	Number of Experts	Average Concordance Value
	**Overexploitation**	**3.68**	14	0.47
*Acacia macrostachya* Rchb. ex DC.	Overgrazing	2.33		
	Fire	2.94		
	Overexploitation	2.93	15	0.54
*Acacia senegal* (L.) Willd	**Overgrazing**	**3.73**		
	Fire	2.87		
	**Overexploitation**	**4.2**	14	0.55
*Adansonia digitata* L.	Overgrazing	2.17		
	Fire	2.43		
	Overexploitation	2.47	14	0.76
*Annona senegalensis* Pers.	Overgrazing	1.66		
	**Fire**	**3.03**		
	Overexploitation	3.29	13	0.49
*Balanites aegyptiaca* (L.) Delile	**Overgrazing**	**3.8**		
	Fire	2.31		
	**Overexploitation**	**4.55**	14	0.63
*Bombax costatum* Pellegr. & Vuill.	Overgrazing	2.13		
	Fire	2.8		
	**Overexploitation**	**2.82**	14	0.54
*Boscia senegalensis* (Pers.) Lam.	Overgrazing	2.57		
	Fire	2.43		
	**Overexploitation**	**3.75**	14	0.63
*Detarium microcarpum* Guill. & Perr.	Overgrazing	1.57		
	Fire	3.06		
	**Overexploitation**	**2.93**	12	0.65
*Lannea microcarpa* Engl. & K. Krause	Overgrazing	2.07		
	Fire	2.85		
	**Overexploitation**	**4.16**	14	0.76
*Parkia biglobosa* (Jacq.) G. Don	Overgrazing	1.92		
	Fire	3.07		
	**Overexploitation**	**2.8**	12	0.33
*Sclerocarya birrea* (A. Rich.) Hochst.	Overgrazing	1.89		
	Fire	2.18		
	Overexploitation	2.19	13	0.56
*Strychnos spinosa* Lam.	Overgrazing	1.91		
	**Fire**	**3.08**		
	Overexploitation	2.98	13	0.65
*Tamarindus indica* L.	Overgrazing	1.67		
	**Fire**	**3.02**		
	**Overexploitation**	**4.16**	14	0.71
*Vitellaria paradoxa* C. F. Gaertn.	Overgrazing	2.02		
	Fire	2.77		
	Overexploitation	2.13	14	0.77
*Ximenia americana* L.	Overgrazing	1.54		
	**Fire**	**2.88**		
	Overexploitation	2.07	14	0.50
*Ziziphus mauritiana* Lam.	Overgrazing	2.84		
	**Fire**	**3.26**		

The greatest threat for each species, marked in bold, is selected based on the highest weighted score. Weighted score values can vary between 1 and 5. The table further shows the number of valid expert responses (number of experts) and the average expert concordance (average concordance value) per species.

To facilitate calculations, the threat sensitivity scores were normalized to obtain values between 0 and 1. Then, the normalized threat sensitivity scores were multiplied by the normalized threat intensity values on a pixel-by-pixel basis. The resulting values were transformed into threat levels as described above.

### Individual and combined threat levels

Six individual threat maps, at a spatial resolution of 2.5 arc-minutes (about 4.5 km at the equator), were created and then aggregated into one combined threat map for each species. The original raster files used to create all the threat maps were made publicly available (https://dataverse.harvard.edu/dataset.xhtml?persistentId=10.7910/DVN/3BTC8J).

The combined threat level of an area, corresponding to an individual pixel, was set to be equal to the highest threat level among the six individual layers. In addition, the threat level of the layer resulting from combining all threats was adjusted upwards if the criteria for the ‘3–5 rule’ were met [54,57] as follows:

- three or more individual threats with threat level ‘High’ are equivalent to threat level ‘Very high’,
- five or more individual threats with threat level ‘Medium’ are equivalent to threat level ‘High’.

It is important to note that threat classes generated through this process do not represent an absolute measure of the impact on food trees but rather the relative degree to which the species are more likely to survive in one place over another based on the threat level of one or more threats, using a common scale for all species.

## Results and discussion

### Patterns of threat magnitude

**Fig 1:**
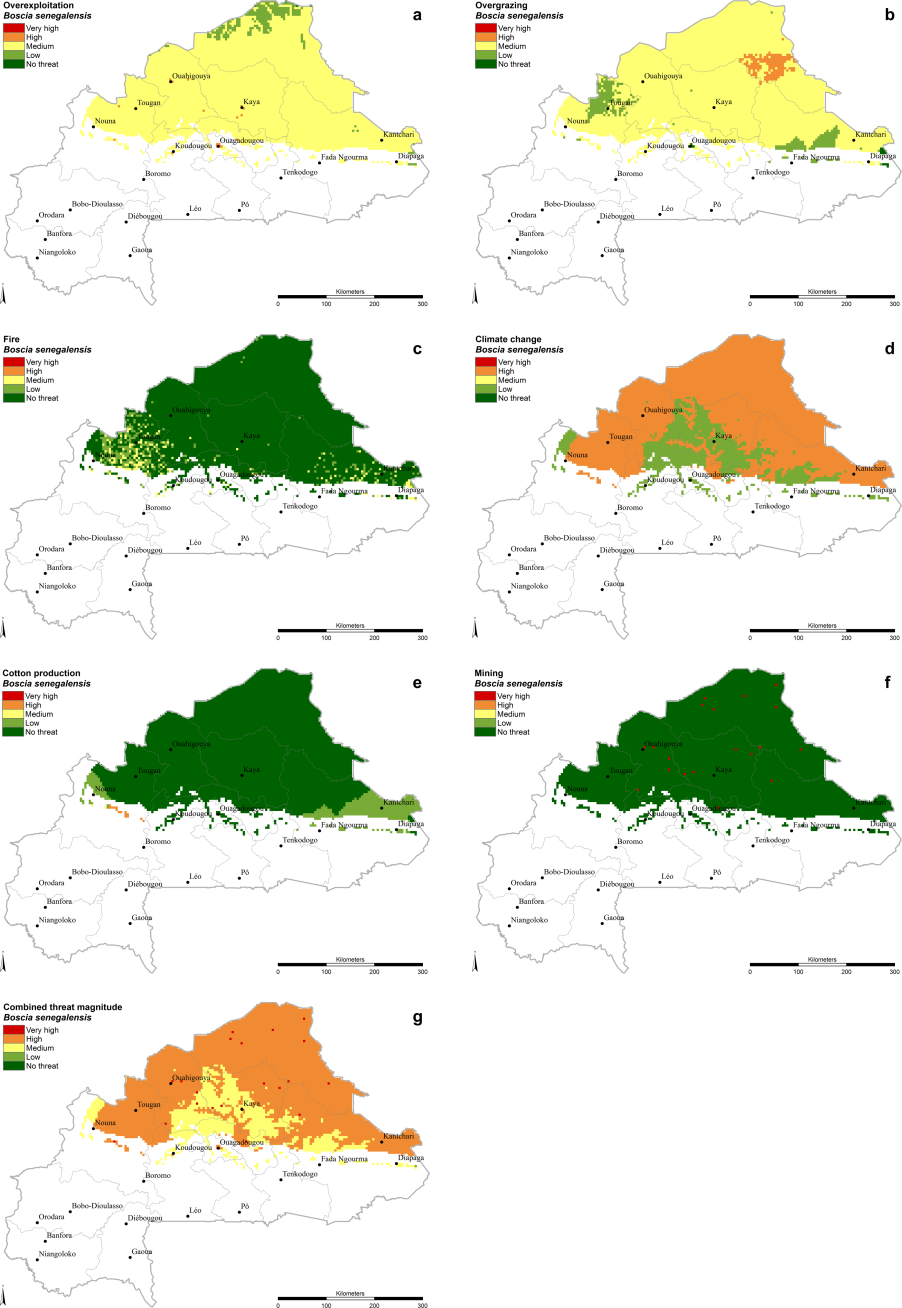
*Boscia senegalensis*. **Threat magnitude levels of (A) ‘Overexploitation’, (B) ‘Overgrazing’, (C) ‘Fire’, (D) ‘Climate change’, (E) ‘Cotton production’, (F) ‘Mining’ and (G) ‘Combined threat’.** The criteria to define the threat levels are presented in Table 5.

**Fig 2:**
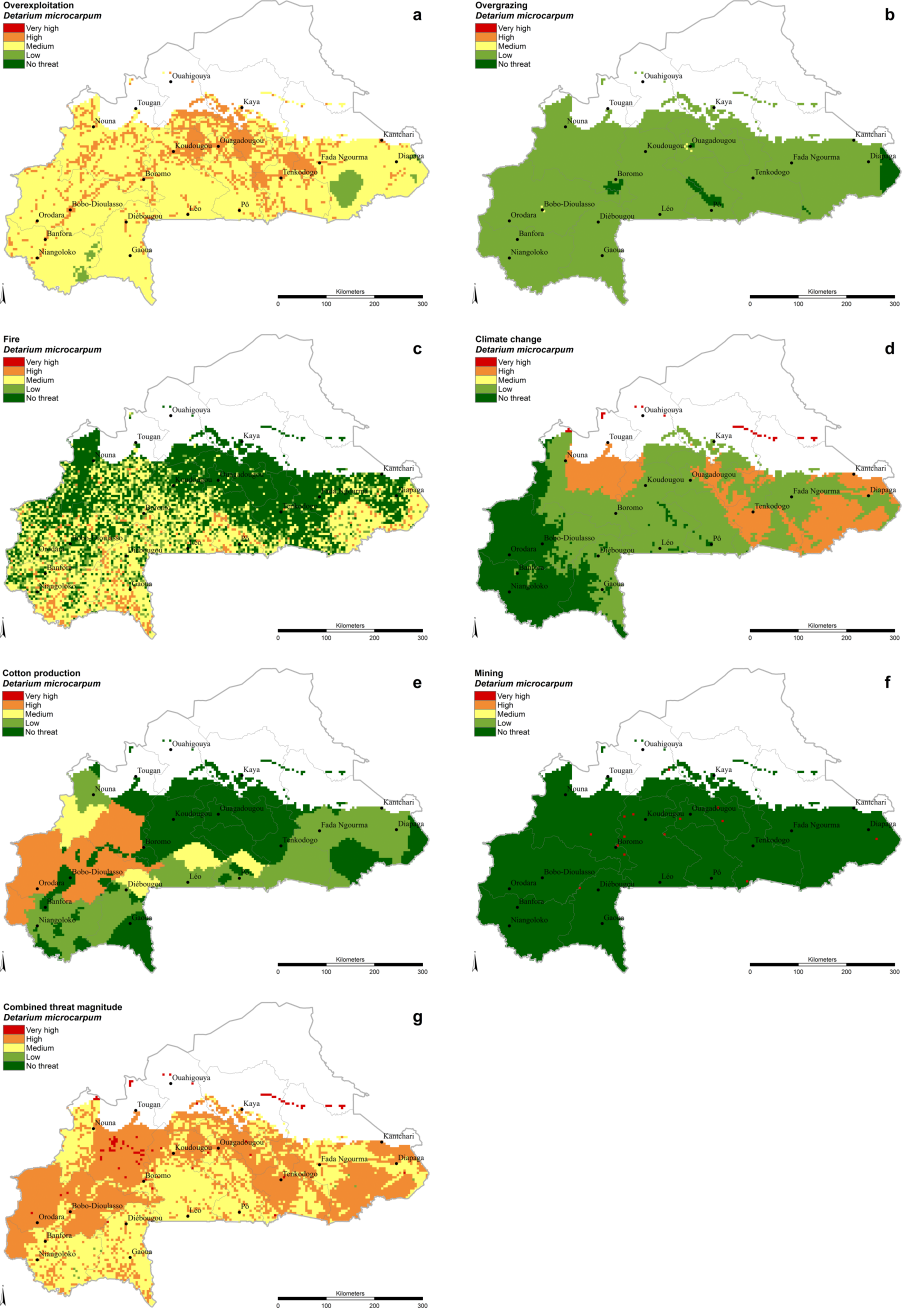
*Detarium microcarpum*. **Threat magnitude levels of (A) ‘Overexploitation’, (B) ‘Overgrazing’, (C) ‘Fire’, (D) ‘Climate change’, (E) ‘Cotton production’, (F) ‘Mining’ and (G) ‘Combined threat’.** The criteria to define the threat levels are presented in Table 5.

The most appropriate distribution map for each species was selected based on the expert survey results; these are presented in Table 2. The final threat magnitude levels attributed to the six main threat factors examined are shown in Table 5. In the case of the species specific threats overexploitation, overgrazing and fire, the sensitivity defined by experts is presented in Table 4. The combination of species distribution models and threat maps resulted in a visualization of species-specific patterns of pressure from threats (presented individually and combined) throughout the distribution range of all 16 selected food tree species within the boundaries of Burkina Faso (Figs 1 and 2, S1–S14 Figs).

**Table 5:** Threat magnitude rating.

Threat magnitude	Overexploitation	Overgrazing	Fire	Cotton production	Mining	Climate change
**Very high**	0.71–1	0.71–1	0.71–1	NA	Presence of mining site (incl. surrounding areas)	One or both scenarios (RCP 4.5 a/o 8.5) predict absence
**High**	0.31–0.7	0.31–0.7	0.31–0.7	0.31–0.7		Both scenarios (RCP 4.5 and 8.5) predict presence in novel regional climate conditions
**Medium**	0.11–0.3	0.11–0.3	0.11–0.3	0.11–0.3		Only scenario RCP 4.5 predicts presence in novel regional climate conditions
**Low**	0.01–0.1	0.01–0.1	0.01–0.1	0.01–0.1		Only scenario RCP 4.5 predicts presence
**No threat**	≤ 0.01	≤ 0.01	≤ 0.01	≤ 0.01	Absence of mining site	Both scenarios (RCP 4.5 and 8.5) predict presence

Threat levels and applied criteria to transform the threat intensity into threat magnitude.

The moderate average concordance rates across species in both expert evaluation surveys may have various reasons and do not necessarily imply a moderate trustworthiness in the results [42]. Apart from differences in knowledge, a diverging interpretation of the survey questions, especially regarding the more abstract concept of threat sensitivity, could also have influenced the results. Although we see clear and reasonable trends in the threat magnitude rating we recognize that there is some arbitrariness in the choice of the cut-off values that requires further investigation. Specific field experiments can provide further insight into the threat intensity-impact relationship such as grazing impact on tree regeneration. Provenance trials may be used to assess the adaptive ability of species to climate change. Assessing land degradation or tree cover change through high resolution satellite imagery and comparing the results with the modeled threat impact is certainly one of the most promising approaches to be tested. Its limitations will likely be the difficulty in clearly associating the detected changes to single tree species and to a single underlying threat.

**Fig 3:**
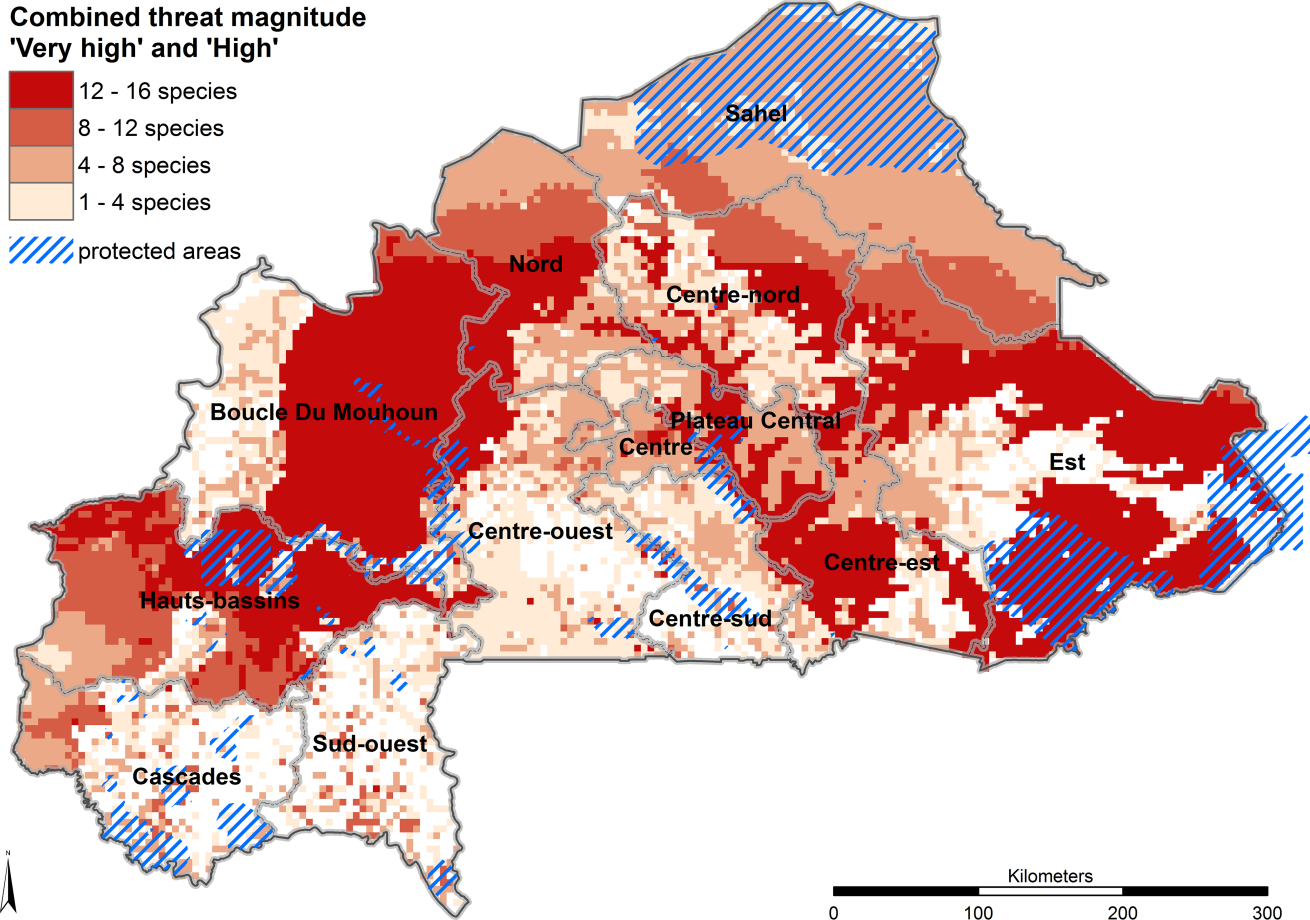
Combined threat magnitude levels ‘Very high’ and ‘High’ for all species across all threats and protected areas. The protected area layer was derived from the World Database on Protected Areas [58]. The criteria to define the threat levels are presented in Table 5.

In order to derive a synthetic index of threat hotspots, a final map was produced combining layers with ‘High’ and ‘Very high’ threat levels (further referred to as ‘severe threat level’ in this study) for all 16 important food tree species (Fig 3).

**Fig 4:**
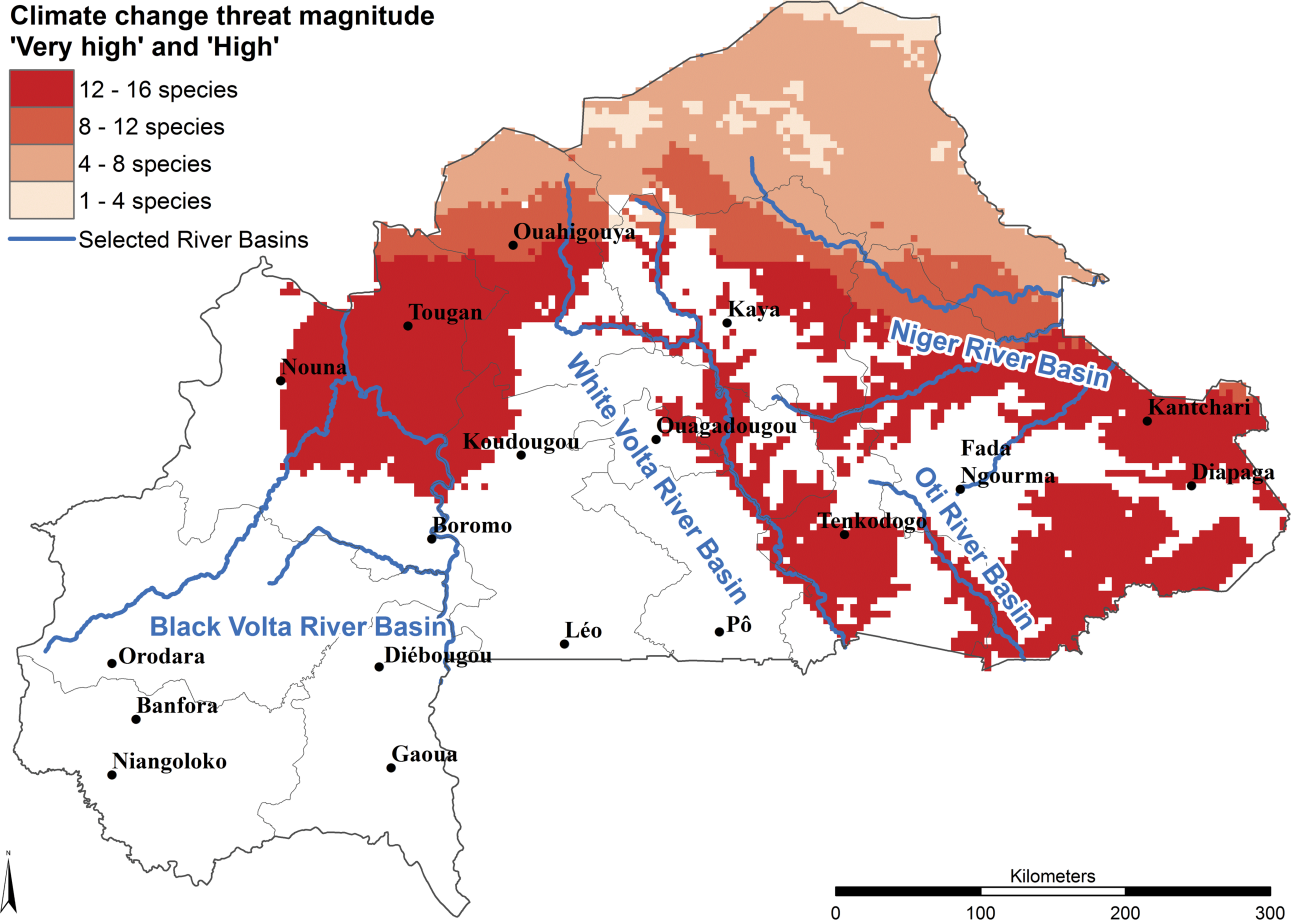
Climate change threat magnitude levels ‘Very high’ and ‘High’ combined for all species. The criteria to define the threat levels are presented in Table 5.

Driven by the findings of previous studies [18,19], suggesting that the progressive tree density and species decline in the anthropogenic-shaped African Sahel over the last 60 years is attributable to climate change, we gave relatively high weight to the predicted 2055 climate change impact on suitable habitat of our target species (Table 5). Under this method, climate change was the most prevalent threat in the long term (according to Table 6) for 13 out of 16 selected food tree species and ‘severe threat levels’ are predicted for an average of 40.5% of the distribution area of the group of study species. The amount of area within a species’ distribution categorized as having a severe threat level reaches a maximum of 78.3% for *Boscia senegalensis* (Fig 1D) because its main distribution area is in the Sahelian zone, the zone most affected by climate change. ‘Very high’ threat levels, where the moderate climate change scenario for 2055 (RCP 4.5) already predicts unsuitable habitat, can be found for *Vitellaria paradoxa* at the northern boundaries of its distribution range affecting 2.9% of the distribution in the country (S12 Fig). The areas with ‘high’ threat level cover 90% of the Sahelian zone (Sahel, the northern parts of Boucle du Mouhoun, Nord, Centre-Nord and Est), about half of the Sudano-Sahelian zone (Central part of Boucle du Mouhoun, Plateau-Central, Centre-Est and Est) including its national reserves in the south but only very small parts of the Sudanian zone in the southeast of Burkina Faso. The areas with ‘severe threat level’ follow the course of some main river basins (White Volta, Niger and Oti River basins) in the central and south-eastern part of the country (Fig 4). The species less affected by climate change are *Detarium microcarpum* (21.8%), *Annona senegalensis* (24.0%) and *Strychnos spinosa* (28.0%) because their occurrence is limited to the wetter areas in the central and southern part of the country, where climate change is expected to have less impact.

**Table 6:** Importance of individual threat layers by species.

species	Overexploitation	Overgrazing	Fire	Cotton production	Mining	Climate change
*Acacia macrostachya*	22.1	0.0	1.5	12.2	0.2	**41.6**
*Acacia senegal*	0.9	19.5	1.0	7.6	0.3	**50.9**
*Adansonia digitata*	38.0	0.0	0.2	12.0	0.2	**44.4**
*Annona senegalensis*	0.1	0.0	0.5	17.7	0.2	**24.0**
*Balanites aegyptiaca*	3.3	20.7	0.0	7.0	0.3	**52.1**
*Bombax costatum*	**57.8**	0.0	1.6	14.2	0.2	32.1
*Boscia senegalensis*	0.3	3.0	0.0	0.3	0.3	**78.3**
*Detarium microcarpum*	18.5	0.0	5.0	18.4	0.2	**21.8**
*Lannea microcarpa*	0.9	0.0	1.3	12.3	0.2	**40.2**
*Parkia biglobosa*	**41.2**	0.0	4.2	14.9	0.2	33.1
*Sclerocarya birrea*	0.4	0.0	0.0	10.8	0.2	**44.3**
*Strychnos spinosa*	0.0	0.0	4.7	15.9	0.2	**28.0**
*Tamarindus indica*	1.1	0.0	3.3	12.9	0.2	**40.4**
*Vitellaria paradoxa*	**41.1**	0.0	1.4	13.7	0.2	36.7
*Ximenia americana*	0.0	0.0	2.2	11.8	0.2	**34.9**
*Ziziphus mauritiana*	0.0	0.4	4.9	10.5	0.2	**46.2**
average	14.1	2.7	2.0	12.0	0.2	**40.5**

Percentage of distribution area (calculated for each species separately) assigned to the six different threats with ‘Very high’ and ‘High’ threat magnitude under this method. The most prevalent threat for each species is highlighted in bold.

We created the climate change threat maps assuming that all selected species react in the same way to climatic conditions becoming less suitable. Also differences in plasticity of functional traits [59], in genetic variability [60], in seed dispersal [61], etc., can play an important role in vulnerability of species to a rapidly changing environment. Due to the existing complexity of the multi-threat approach, we decided not to consider this additional aspect in our analysis.

Based on the average proportion of distribution area affected by a ‘severe threat level’ for all species, overexploitation emerged as the second most important threat in general, and as the most important short-term threat. It is the single most important threat for *Bombax costatum* (57.8%), *Parkia biglobosa* (41.2%) and *Vitellaria paradoxa* (41.1%), and is only slightly exceeded by climate change in the case of *Adansonia digitata* (38.0%) (Table 6). Areas with a ‘Very high’ level of threat from overexploitation in Burkina Faso can be found in more or less close vicinity to the largest cities (Ouagadougou, Bobo-Dioulasso, Koudougou and Banfora) but also near smaller populated places (e.g. Kaya, Ouahigouya, Tougan, Tenkodogo, and Léo) well connected to the road network. The main regions with a ‘severe threat level’ due to overexploitation are Centre, Centre-Sud, Plateau-Central, Centre-Nord, Nord and Boucle du Mouhoun.

In our analysis we used one single spatial layer, the Global Human Footprint, as a proxy for the different aspects of overexploitation (such as harvesting for food, timber, fuelwood, and animal fodder). Although it combines various anthropogenic factors likely to have an impact on the environment, we are aware that the aspects of overexploitation follow different dynamics that might not be equally related to the intensity of human influence.

Cotton production represents a threat for those tree species that occur in the intensive cotton production areas. In Burkina Faso, the area of cotton production is characterized by relatively abundant rainfalls of 800-1000mm/a [15]. The species most affected is *Detarium microcarpum* (Fig 2E), with 18.4% of its distribution area under high threat from competition with cotton production. The species least threatened in this analysis is *Boscia senegalensis* (Fig 1E) with only 0.3% of its distribution area affected. Large areas with a ‘High’ threat level can be found in Hauts-Bassins, Boucle du Mouhoun and the north-western part of the Cascades region excluding the designated National Classified Forest areas (Maro, Tui, Kapo, Tere, Pa and Toroba).

We selected cotton production instead of agricultural production in general as the main threat to food tree species because of its particular importance in Burkina Faso. It is considered the principal source of income for rural populations and the nearly threefold increase in cotton production between 1995 and 2005 was achieved mainly due to expansion of cultivated land [49]. Due to the lack of precise cotton production intensity maps, we assigned the maximum threat level as ‘High’ which is likely to underestimate the actual threat potential posed to food tree species. As we had assumed easy access to high resolution maps developed by local authorities, we did not explore the potential of directly assessing production intensity from satellite imagery. After various unsuccessful attempts with local authorities, we found a viable compromise by georeferencing a production area map and combining it with provincial production statistics.

Overgrazing is estimated to be the fourth most prevalent threat (Table 6) but with a larger impact in the Sahelian zone, in the northern part of Burkina Faso, a traditional herding area. It is the second most important threat for *Balanites aegyptiaca* (20.7%), *Acacia senegal* (19.5%) and *Boscia senegalensis* (3.0%) following climate change. ‘High’ threat levels can be found in the Sahel, Centre, Plateau-Central, in the northern part of Est and Centre-Est and in some parts of the Centre-Ouest and Hauts-Bassins regions. Based on availability we combined cattle, goat and sheep density to create TLUs to account for different amounts of feed. Additional information on distinct grazing preferences and livestock management systems would be needed to make more precise predictions of livestock pressure on tree regeneration. Tree-regeneration projects in Sahelian West Africa have demonstrated success through specific protection of seedlings from grazing and through selective clearing of fields. The farmers promoting assisted regeneration of natural woody species benefited from sufficient wood production for their household and increasing sustainable crop yields after 2–3 years [62].

Fire is the second least prevalent threat in general (Table 6), and ‘High’ threat levels occur only in 2%, on average, of the distribution area of all tree species analyzed. Some species emerge to be more vulnerable to fire because of the higher share of distribution in fire frequent areas and a higher threat sensitivity rating (Table 4), although experts noted only little difference among species: *Detarium microcarpum* (5.0%), *Ziziphus mauritiana* (4.9%) *and Strychnos spinosa* (4.7%). High levels of threat from fire are common in the southern regions, such as Cascades Sud-Ouest, Centre-Sud and Est, especially in the reserves and national parks (e.g. Kabore-Tambi, Singou, Pama, Arly and ‘W’ Region Biosphere Reserve) due to the thick grasslands that characterize these areas. The vulnerability of species to fire, especially in the seedling stage, depends very much on fire frequency. Fire frequency was calculated from single fire locations deriving from a reliable data source, FIRMS, which provided remotely-sensed information on fire events in Burkina Faso between 2007 and 2012.

Mining is the least prevalent threat taking into account the average distribution area where ‘severe threat levels’ occur for all selected species (Table 6). Although of minor relevance for food trees at the country level, in mining sites and surrounding areas serious habitat destruction and degradation occur at a local scale. The continuing boom in gold production and exploration activities might further increase the local threats to food tree species. Most of the current mining activities, primarily gold mining, are located in the Sahel, Nord, Centre-Nord and in the northern part of the Centre-Ouest region.

### Combination of threats

All 16 selected food tree species are highly threatened over large areas of their distribution in Burkina Faso (ranging from 45 to 78% of the area); on average 60.5% of the distribution area of these tree species is highly threatened. Only a small area is classified as ‘very highly’ threatened (average 0.7%—see Table 7). Protected areas such as faunal reserves, classified forests and national parks in the Est, Boucle du Mouhoun and Hauts-Bassins regions (Fig 3) may not afford protection to species that are highly threatened throughout much of their distribution area. The highest threats are mainly from climate change and fire whereas establishing protected areas potentially protects against overexploitation, cotton production, overgrazing and mining.

**Table 7:** Combined threat layers by species.

Species	Very high	High	Medium	Low	No threat
*Boscia senegalensis*	0.4	**78.1**	21.5	0	0
*Bombax costatum*	**1.5**	**73.9**	24.5	0	0
*Adansonia digitata*	0.7	**72.7**	26.5	0.1	0
*Parkia biglobosa*	0.8	69.7	29.4	0.1	0
*Vitellaria paradoxa*	**3.6**	66.6	29.8	0.1	0
*Balanites aegyptiaca*	0.9	67.2	31.9	0.1	0
*Acacia senegal*	0.5	66.1	33.3	0	0
*Acacia macrostachya*	0.5	64.1	35.3	0.1	0
*Ziziphus mauritiana*	0.3	59.1	39.8	0.8	0
*Tamarindus indica*	0.2	55.1	44.4	0.3	0
*Detarium microcarpum*	**1.0**	53.6	45.2	0.1	0
*Sclerocarya birrea*	0.3	53.6	45.7	0.4	0
*Lannea microcarpa*	0.3	52.4	**47.2**	0.2	0
*Ximenia americana*	0.2	46.9	44.8	**8**	0
*Strychnos spinosa*	0.2	45.8	**48.2**	**5.7**	0
*Annona senegalensis*	0.2	43.5	**54.4**	**1.9**	0
average	0.7	60.5	37.6	1.1	0

Percentage of distribution area (calculated for each species separately) assigned to the five different threat levels (‘Very high’ to ‘No threat’) under this method. The three highest percentages per threat level are highlighted in bold. The species are ranked based on the percentage of distribution area under ‘severe threat’ (‘High’ and ‘Very high’ threat level).

The species for which the highest percentages of distribution area are under ‘severe threat’ are *Boscia senegalensis* (78.5%), *Bombax costatum* (75.4%) and *Adansonia digitata* (73.4%). The high threat status is caused nearly exclusively by climate change (Table 7) in the case of *Boscia senegalensis* and by the combination of overexploitation and climate change in the cases of *Bombax costatum* and *Adansonia digitata* (for spatial details see Fig 1, S3 and S6 Figs). *Parkia biglobosa*, a multi-purpose tree species widely used by local communities ranks fourth (Table 7), followed by the most abundant tree species in agroforestry parklands in the Sudanian zone, *Vitellaria paradoxa*. Both *Parkia biglobosa* and *Vitellaria paradoxa* show ‘severe threat levels’ caused mainly by overexploitation, climate change and cotton production (Table 7). The species with the lowest percentage of distribution area under ‘severe threat’ is *Annona senegalensis* (43.7%), partly because it occurs in areas where climate change is expected to have less impact (S4 Fig) and partly because of its lower threat sensitivity (Table 4).

**Fig 5:**
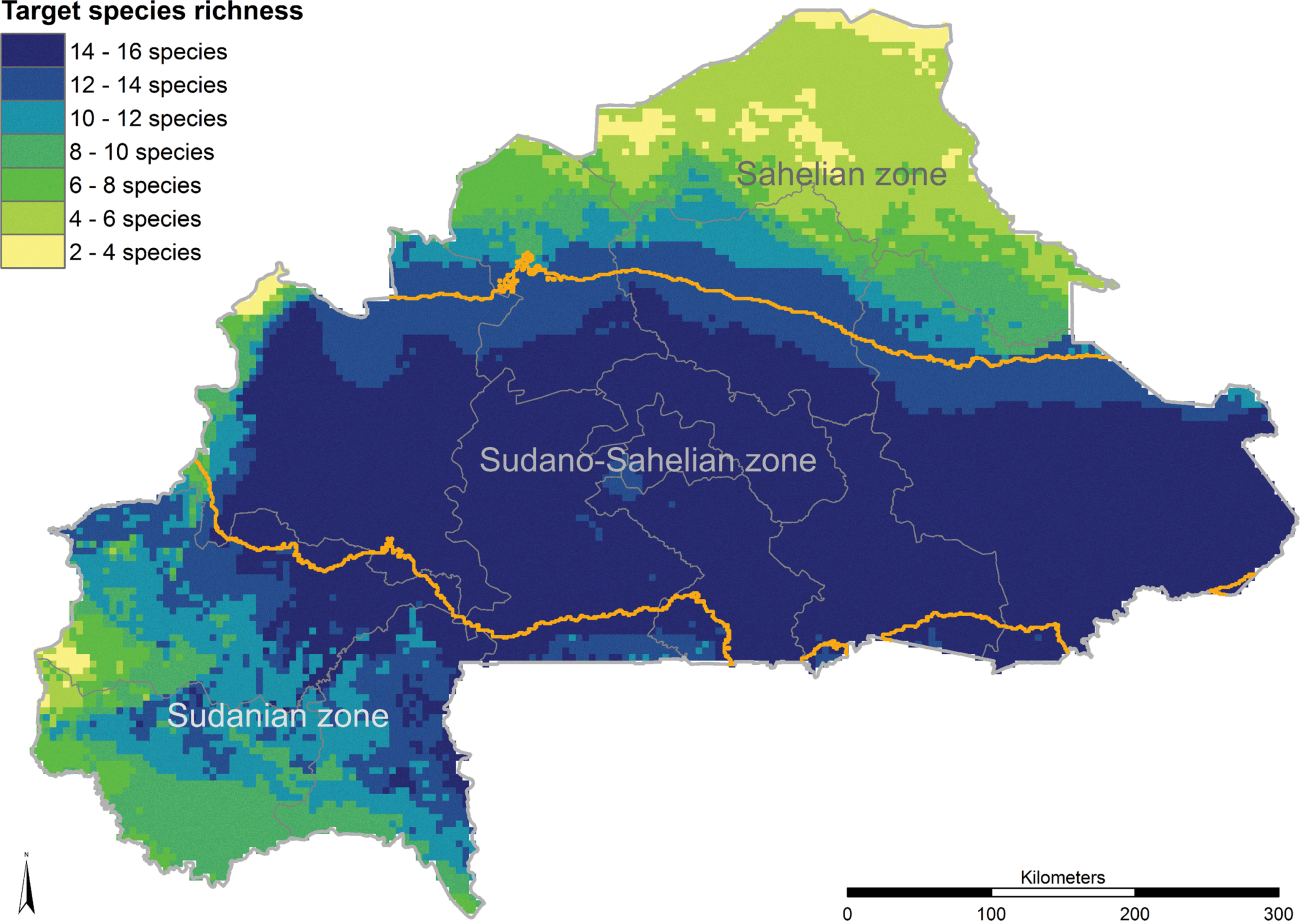
Species richness map of 16 important food tree species and eco-climatic zones. The three eco-climatic zones (Sahelian zone: < 600mm/a, Sudano-Sahelian zone: 600–900 mm/a and Sudanian zone: > 900 mm/a) are defined by the annual rainfall [4] and are represented in this map by the bioclimatic variable 12 from the WorldClim 1.4 dataset [41].

The highest species richness of the 16 target species in this study, determined on the basis of SDMs predicting presence of suitable habitat, can be found in the central Sudano-Sahelian and the northern Sudanian zone (Fig 5) and the largest areas that are severely threatened for all species combined occur in the central-eastern parts of Boucle du Mouhon (Nayala, Mouhon, and Kossi provinces), in the northern part of Centre-Ouest (Sanguie province), in parts of Plateau-Central and Centre-Est and in the south-eastern parts of Est (Tapoa and Kopienga provinces) (Fig 3). The main threats in Boucle du Mouhon are cotton production, climate change and overexploitation while the central and southern regions are mostly affected by climate change and partly also by fire.

## Conclusions and recommendations

The species-level spatial threat analysis presented in this study identifies critical areas for conservation of populations of important food tree species in Burkina Faso. The multi-threat approach facilitates determining which threat contributes most to high threat levels in certain areas of the country, which is crucial for specific decisions on conservation and management actions.

The approach can be easily transferred to other countries and can be used to analyze general and species specific threats at finer and more local as well as at broader (continental) scales. The concept can be applied anywhere as long as appropriate spatial data are available as well as knowledgeable experts. Expert input is crucial to refine the species distribution as well as the magnitude of the threats. We learned that an online survey can be a valid alternative to conducting workshops, having the advantage of being less time- and cost-intensive. The model is repeatable and it could be applied again in the future to determine if threat levels for certain species and populations have increased or decreased in specific areas.

The results of this study indicate that all 16 species face serious threats throughout much of their distribution in Burkina Faso and that climate change is predicted to be the most prevalent threat in the long term. Overexploitation is the most important short-term threat at the national level, although cotton production is a more serious threat in the region where it is grown. The predicted decline of habitat suitability together with evidence from other studies [18,19] of natural regeneration problems due to climate change and human population strongly suggest the need for conservation measures. We recommend that tree populations growing in areas designated as ‘severely’ threatened (Fig 4, Figs 1D and 2D, S1–S14 Figs) due to climate change should be used as seed sources for *ex situ* conservation and planted in areas where predicted future climate would produce suitable habitat. Assisted regeneration is suggested for populations growing in areas where habitat is predicted under future climate conditions, coinciding with a ‘severe’ threat level due to short-term threats such as overexploitation and/or overgrazing.

More than 55% of the distribution of ten of the species is under high or very high threat. Conservation planning is urgent for these species and plans should be developed to focus on the specific threats to targeted populations, prioritizing the species and if possible, the populations, that are most important to local people. For example, *Vitellaria paradoxa* is very highly threatened by climate change along its northern margin. Valuable seed sources in this area may be lost unless seed is collected for planting in more suitable climate and/or for *ex situ* conservation. Populations highly threatened by overexploitation in the central part of Burkina Faso, between Ouagadougou, Kadougou and Kaya, should be prioritized for assisted regeneration as they grow in areas where predicted future climate would produce suitable habitat. *Parkia biglobosa*, another highly valuable species, is also highly threatened by a combination of climate change, overexploitation and cotton production across most of its distribution. The recommendations on population level regarding conservation measures for *Vitellaria paradoxa* and *Parkia biglobosa* are very similar. Both species are highly threatened near the northern limit of intensive cotton production. Knowing the regions where the various threats are most serious allows targeting actions to address the particular threats at the population level (S8 and S12 Figs) so that government agencies can take specific conservation actions to maintain the genetic diversity across the species’ distribution range. In the same way, population specific recommendations can be derived from the individual and combined threat maps of the other selected food tree species.

The visual and spatially explicit representation of the threats and their predicted impact, in the form of maps with different threat levels, makes the results easily accessible and understandable to decision makers from private and public agencies. Identifying areas with high threat levels for certain species and knowing the underlying cause can guide specific recommendations on priority areas for the implementation of conservation efforts.

This analysis represents one step towards a detailed understanding of multi-species-threat relations across time, and studies should follow aiming to refine and test additional methodologies, such as remote sensing to measure threat intensities and for pseudo ground truthing or provenance trials to assess climate change impacts. Spatial and temporal resolution of information is continuously increasing and will lead to more precise predictions and monitoring of areas likely to become severely threatened in order to plan more selective and efficient conservation actions in time.

## Supporting information

S1 Dataset Georeferenced tree distribution records. (XLSX)

S1 Appendix Online survey questions. (PDF)

S1 Fig *Acacia macrostachya*. Threat magnitude levels of (a) ‘Overexploitation’, (B) ‘Overgrazing’, (C) ‘Fire’, (D) ‘Climate change’, (E) ‘Cotton production’, (F) ‘Mining’ and (G) ‘Combined threat’.
(TIF)

S2 Fig *Acacia senegal*. Threat magnitude levels of (A) ‘Overexploitation’, (B) ‘Overgrazing’, (C) ‘Fire’, (D) ‘Climate change’, (E) ‘Cotton production’, (F) ‘Mining’ and (G) ‘Combined threat’.
(TIF)

S3 Fig *Adansonia digitata*. Threat magnitude levels of (A) ‘Overexploitation’, (B) ‘Overgrazing’, (C) ‘Fire’, (D) ‘Climate change’, (E) ‘Cotton production’, (F) ‘Mining’ and (G) ‘Combined threat’.
(TIF)

S4 Fig *Annona senegalensis*. Threat magnitude levels of (A) ‘Overexploitation’, (B) ‘Overgrazing’, (C) ‘Fire’, (D) ‘Climate change’, (E) ‘Cotton production’, (F) ‘Mining’ and (G) ‘Combined threat’.
(TIF)

S5 Fig *Balanites aegyptiaca*. Threat magnitude levels of (A) ‘Overexploitation’, (B) ‘Overgrazing’, (C) ‘Fire’, (D) ‘Climate change’, (E) ‘Cotton production’, (F) ‘Mining’ and (G) ‘Combined threat’.
(TIF)

S6 Fig *Bombax costatum*. Threat magnitude levels of (A) ‘Overexploitation’, (B) ‘Overgrazing’, (C) ‘Fire’, (D) ‘Climate change’, (E) ‘Cotton production’, (F) ‘Mining’ and (G) ‘Combined threat’.
(TIF)

S7 Fig *Lannea microcarpa*. Threat magnitude levels of (A) ‘Overexploitation’, (B) ‘Overgrazing’, (C) ‘Fire’, (D) ‘Climate change’, (E) ‘Cotton production’, (F) ‘Mining’ and (G) ‘Combined threat’.
(TIF)

S8 Fig *Parkia biglobosa*. Threat magnitude levels of (A) ‘Overexploitation’, (B) ‘Overgrazing’, (C) ‘Fire’, (D) ‘Climate change’, (E) ‘Cotton production’, (F) ‘Mining’ and (G) ‘Combined threat’.
(TIF)

S9 Fig *Sclerocarya birrea*. Threat magnitude levels of (A) ‘Overexploitation’, (B) ‘Overgrazing’, (C) ‘Fire’, (D) ‘Climate change’, (E) ‘Cotton production’, (F) ‘Mining’ and (G) ‘Combined threat’.
(TIF)

S10 Fig *Strychnos spinosa*. Threat magnitude levels of (A) ‘Overexploitation’, (B) ‘Overgrazing’, (C) ‘Fire’, (D) ‘Climate change’, (E) ‘Cotton production’, (F) ‘Mining’ and (G) ‘Combined threat’.
(TIF)

S11 Fig *Tamarindus indica*. Threat magnitude levels of (A) ‘Overexploitation’, (B) ‘Overgrazing’, (C) ‘Fire’, (D) ‘Climate change’, (E) ‘Cotton production’, (F) ‘Mining’ and (G) ‘Combined threat’.
(TIF)

S12 Fig *Vitellaria paradoxa*. Threat magnitude levels of (A) ‘Overexploitation’, (B) ‘Overgrazing’, (C) ‘Fire’, (D) ‘Climate change’, (E) ‘Cotton production’, (F) ‘Mining’ and (G) ‘Combined threat’.
(TIF)

S13 Fig *Ximenia americana*. Threat magnitude levels of (A) ‘Overexploitation’, (B) ‘Overgrazing’, (C) ‘Fire’, (D) ‘Climate change’, (E) ‘Cotton production’, (F) ‘Mining’ and (G) ‘Combined threat’.
(TIF)

S14 Fig *Ziziphus mauritiana*. Threat magnitude levels of (A) ‘Overexploitation’, (B) ‘Overgrazing’, (C) ‘Fire’, (D) ‘Climate change’, (E) ‘Cotton production’, (F) ‘Mining’ and (G) ‘Combined threat’.
(TIF)
