# Correction: Spatially explicit multi-threat assessment of food tree species in Burkina Faso: A fine-scale approach

**DOI:** 10.1371/journal.pone.0190760

**Published:** 2018-01-02

**Authors:** The PLOS ONE Staff

The Data Availability statement for this paper contains an incorrect link. The publisher apologizes for this error. The correct statement is: Data are available within the paper and the Supporting Information files. The associated raster files to create the threat maps (Figs 1–5, S1-S14 Figs) are available on Harvard Dataverse: https://dataverse.harvard.edu/dataset.xhtml?persistentId=doi:10.7910/DVN/3BTC8J
