# Supplementary material for: Spatially explicit multi-threat assessment of food tree species in Burkina Faso: A fine-scale approach
Source: PLoS One. 2017 Sep 7;12(9):e0184457. doi: 10.1371/journal.pone.0184457 (PMC5589249; doi:10.1371/journal.pone.0184457)
Supplement: S1 Appendix — (PDF) [file pone.0184457.s002.pdf]

Online survey questions

Spatial distribution models

A series of maps showing the predicted natural distribution of 16 important tree species were developed based on different parameters included in the models. This survey has the objective to identify for each species the model that best represents its current distribution, through the opinion of experts.

When studying the natural distribution of the species, it is necessary to have sufficient records with valid coordinates, but for some of these species, such records available to the public are limited. To overcome the poor number of records for some species, ecological niche modeling is used, which allows to predict the natural range of each species. This technique creates an ecological niche from the environmental characteristics of each site where a species has been observed and identifies other areas with similar environmental conditions as areas where the species can potentially occur. The advantage of this method is that it can be consistently applied to a large number of species.

Below you will be able to display 4 different ecological niche models (each produced from different sets of environmental variables) for 16 species.

Model 1

In a scale from 1 to 5, assign a value to validity of the model, considering that 1 = not valid, 2 = poor, 3 = regular, 4 = good, 5 = excellent

1	2	3	4	5
○	○	○	○	○

Model 2

In a scale from 1 to 5, assign a value to validity of the model, considering that 1 = not valid, 2 = poor, 3 = regular, 4 = good, 5 = excellent

1	2	3	4	5
○	○	○	○	○

Model 3

In a scale from 1 to 5, assign a value to validity of the model, considering that 1 = not valid, 2 = poor, 3 = regular, 4 = good, 5 = excellent

1	2	3	4	5
○	○	○	○	○

Model 4

In a scale from 1 to 5, assign a value to validity of the model, considering that 1 = not valid, 2 = poor, 3 = regular, 4 = good, 5 = excellent

1	2	3	4	5
○	○	○	○	○

Threat sensitivity

A second objective of the survey is to collect expert knowledge on the sensitivity of each of the 16 species to common agents of pressure that produce changes in the forest cover, and determine the vulnerability of each species to each of these.

The survey includes only the most important threats for which datasets on national level were available to create the threat models. To be able to model potential impact of various threats we would like to introduce a **five-point rating scale** using the following definitions (adapted from TNC 2007):

**Very high:**	The threat is likely to destroy or eliminate the target or reduce its population by 71-100%
**High:**	The threat is likely to seriously degrade the target or reduce its population by 31-70%
**Medium:**	The threat is likely to moderately degrade the target or reduce its population by 11-30%
**Low:**	The threat is likely to only slightly degrade the target or reduce its population by 1-10%
**No threat:**	No threat potential (degrading the population by less than 1%)

Please rate for each species the **sensitivity to a certain threat** by marking with a **cross (X)** the rating that you think is the most appropriate. Or, in other words, please estimate how damaging a threat can be locally to individual populations when it occurs in its highest intensity.

Threat 1: Threats through human access (this includes harvesting for timber, cutting to collect firewood, over-extraction of non-timber forest products such as bark, leave, fruits, etc.)

Target species	Very high	High	Medium	Low	No threat	"Don't know"
Acacia macrostachya						
Acacia senegal						
Adansonia digitata						
Annona senegalensis						
Balanites aegyptiaca						
Bombax costatum						
Boscia senegalensis						
Detarium microcarpum						
Lannea microcarpa						
Parkia biglobosa						
Sclerocarya birrea						
Strychnos spinosa						
Tamarindus indica						
Vitellaria paradoxa						
Ximenia americana						
Ziziphus mauritiana						

Additional comments or more detailed explanations on **Threat 1: Threats through human access**:

Threat 2: Livestock grazing

Target species	Very high	High	Medium	Low	No threat	"Don't know"
Acacia macrostachya						
Acacia senegal						
Adansonia digitata						

Annona senegalensis						
Balanites aegyptiaca						
Bombax costatum						
Boscia senegalensis						
Detarium microcarpum						
Lannea microcarpa						
Parkia biglobosa						
Sclerocarya birrea						
Strychnos spinosa						
Tamarindus indica						
Vitellaria paradoxa						
Ximenia americana						
Ziziphus mauritiana						

Additional comments or more detailed explanations on **Threat 2: Livestock grazing**:

Threat 3: Fire

**Target species**	**Very high**	**High**	**Medium**	**Low**	**No threat**	**“Don’t know”**
Acacia macrostachya						
Acacia senegal						
Adansonia digitata						
Annona senegalensis						
Balanites aegyptiaca						
Bombax costatum						
Boscia senegalensis						
Detarium microcarpum						
Lannea microcarpa						
Parkia biglobosa						
Sclerocarya birrea						
Strychnos spinosa						
Tamarindus indica						
Vitellaria paradoxa						
Ximenia americana						
Ziziphus mauritiana						

Additional comments or more detailed explanations on **Threat 3: Fire**:
