# Supplementary figures and images for: Spatially explicit multi-threat assessment of food tree species in Burkina Faso: A fine-scale approach
Source: PLoS One. 2017 Sep 7;12(9):e0184457. doi: 10.1371/journal.pone.0184457 (PMC5589249; doi:10.1371/journal.pone.0184457)

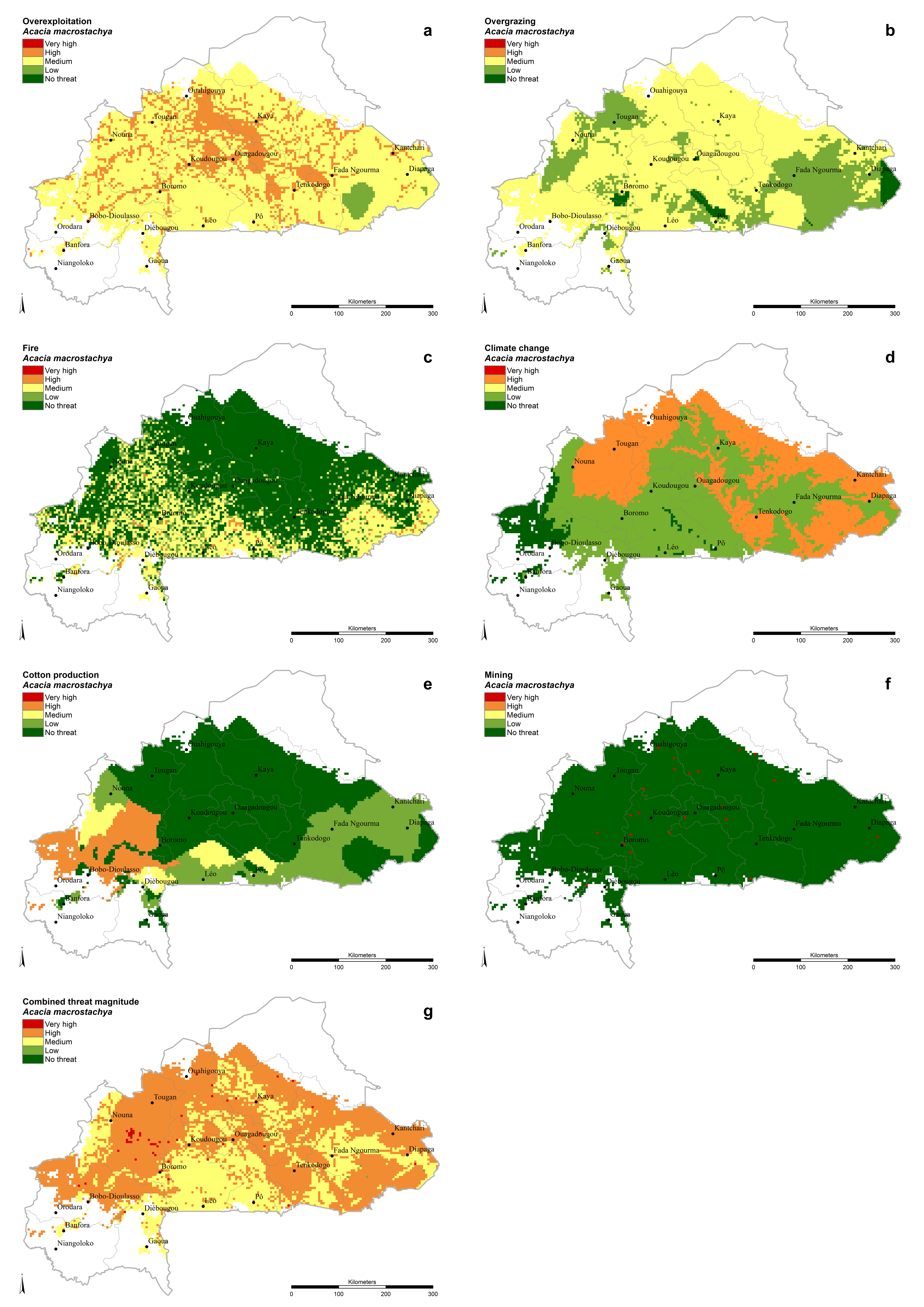

Supplement: S1 Fig — Threat magnitude levels of (a) ‘Overexploitation’, (B) ‘Overgrazing’, (C) ‘Fire’, (D) ‘Climate change’, (E) ‘Cotton production’, (F) ‘Mining’ and (G) ‘Combined threat’. (TIF) [file pone.0184457.s003.tif]

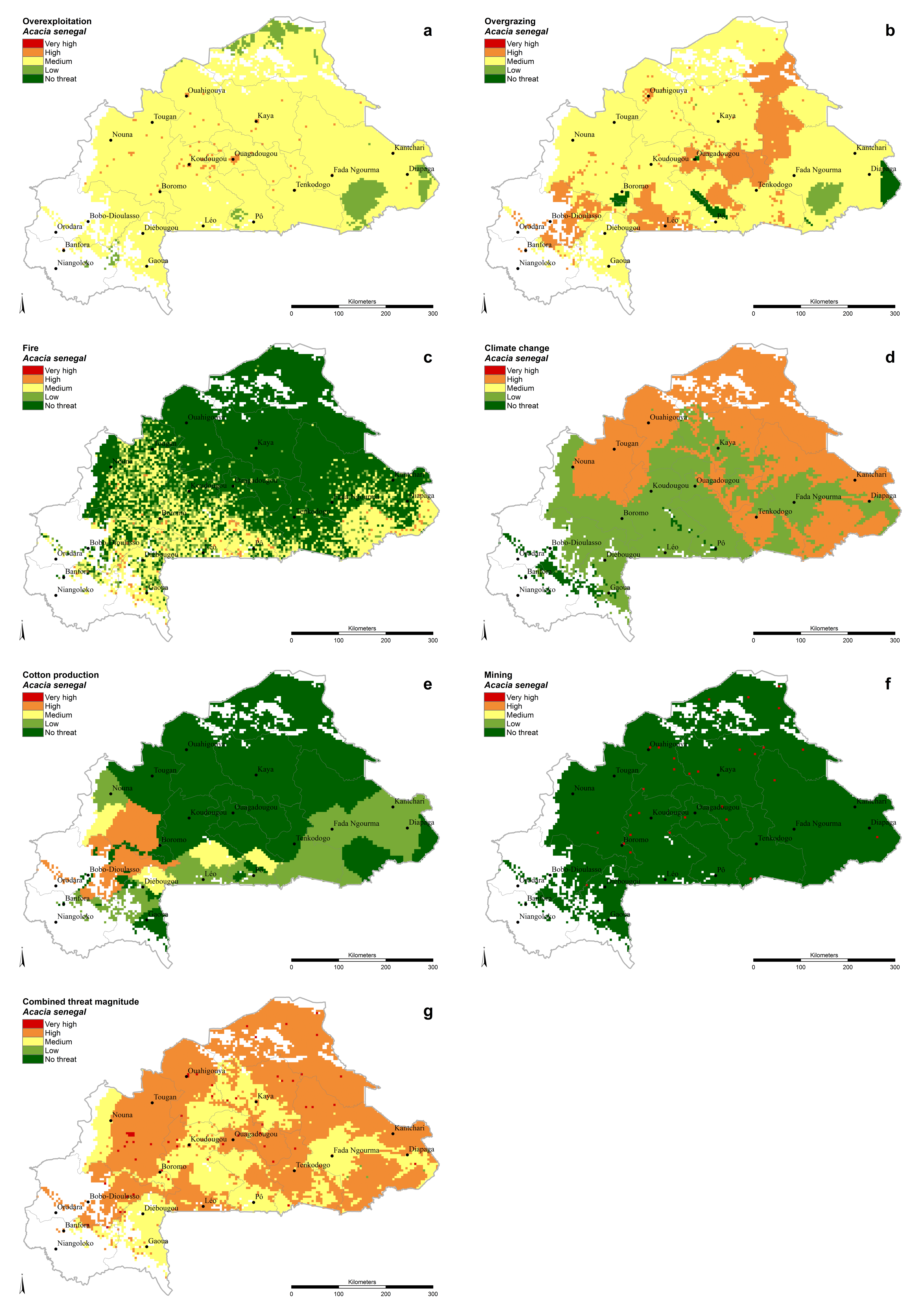

Supplement: S2 Fig — Threat magnitude levels of (A) ‘Overexploitation’, (B) ‘Overgrazing’, (C) ‘Fire’, (D) ‘Climate change’, (E) ‘Cotton production’, (F) ‘Mining’ and (G) ‘Combined threat’. (TIF) [file pone.0184457.s004.tif]

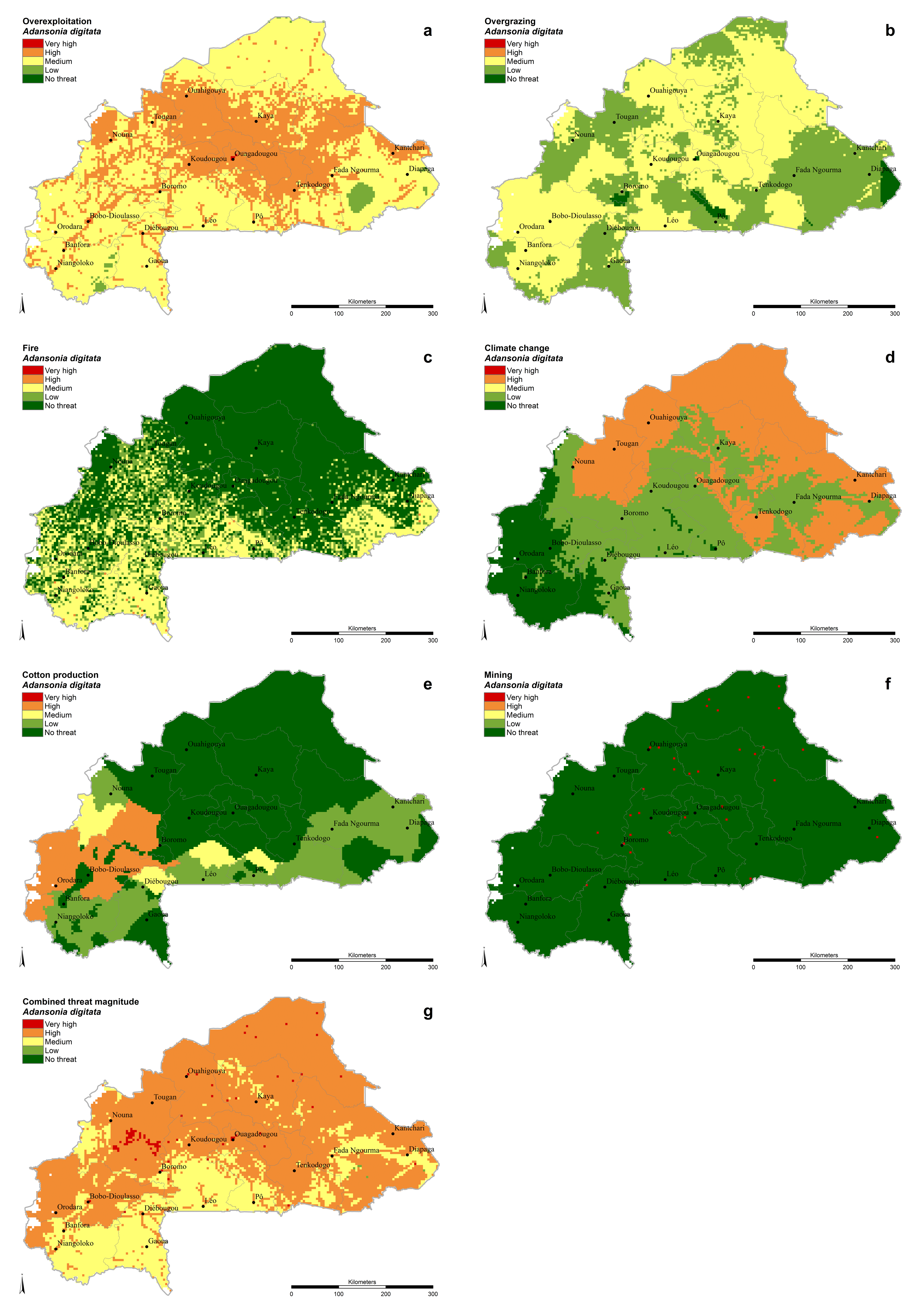

Supplement: S3 Fig — Threat magnitude levels of (A) ‘Overexploitation’, (B) ‘Overgrazing’, (C) ‘Fire’, (D) ‘Climate change’, (E) ‘Cotton production’, (F) ‘Mining’ and (G) ‘Combined threat’. (TIF) [file pone.0184457.s005.tif]

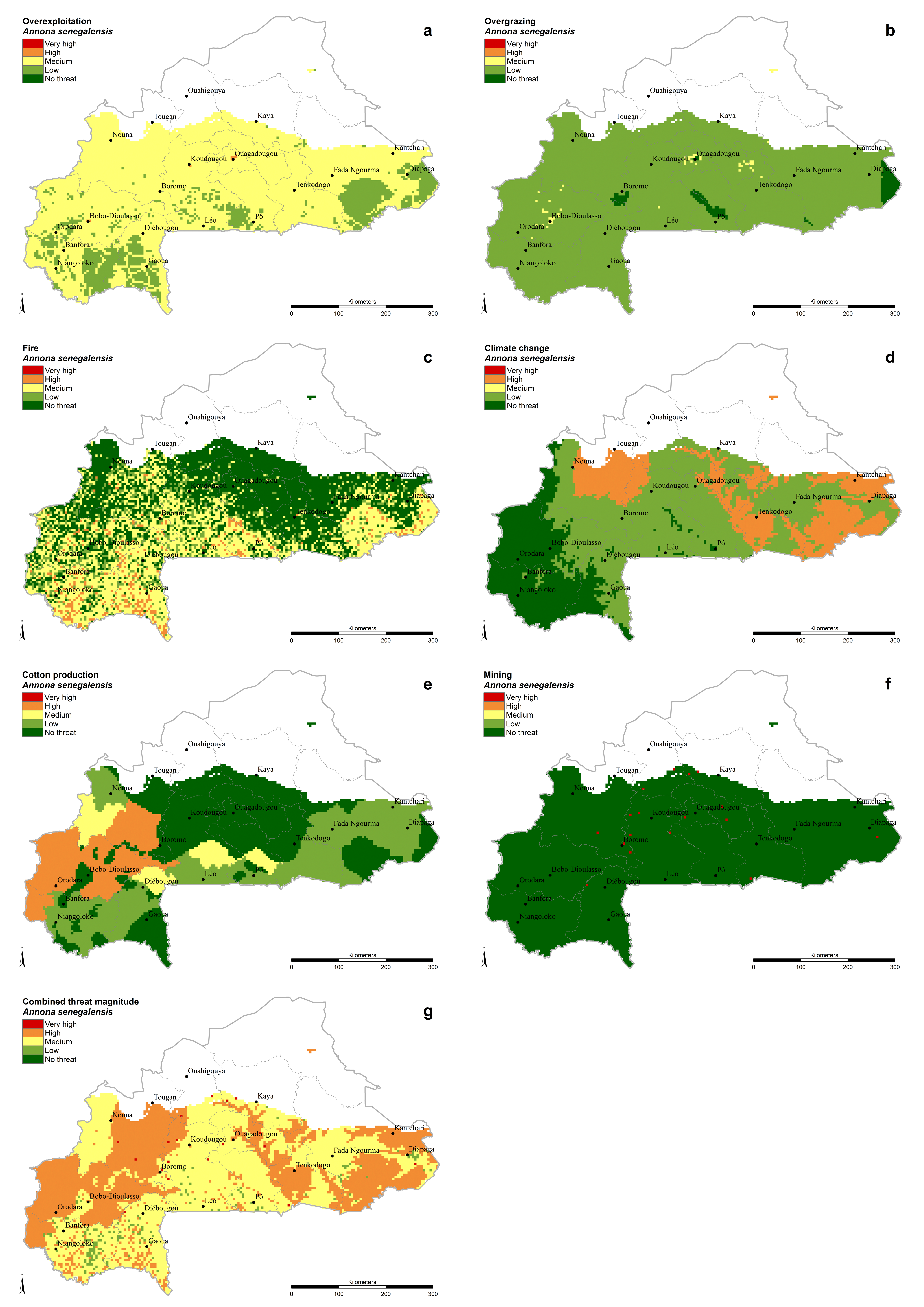

Supplement: S4 Fig — Threat magnitude levels of (A) ‘Overexploitation’, (B) ‘Overgrazing’, (C) ‘Fire’, (D) ‘Climate change’, (E) ‘Cotton production’, (F) ‘Mining’ and (G) ‘Combined threat’. (TIF) [file pone.0184457.s006.tif]

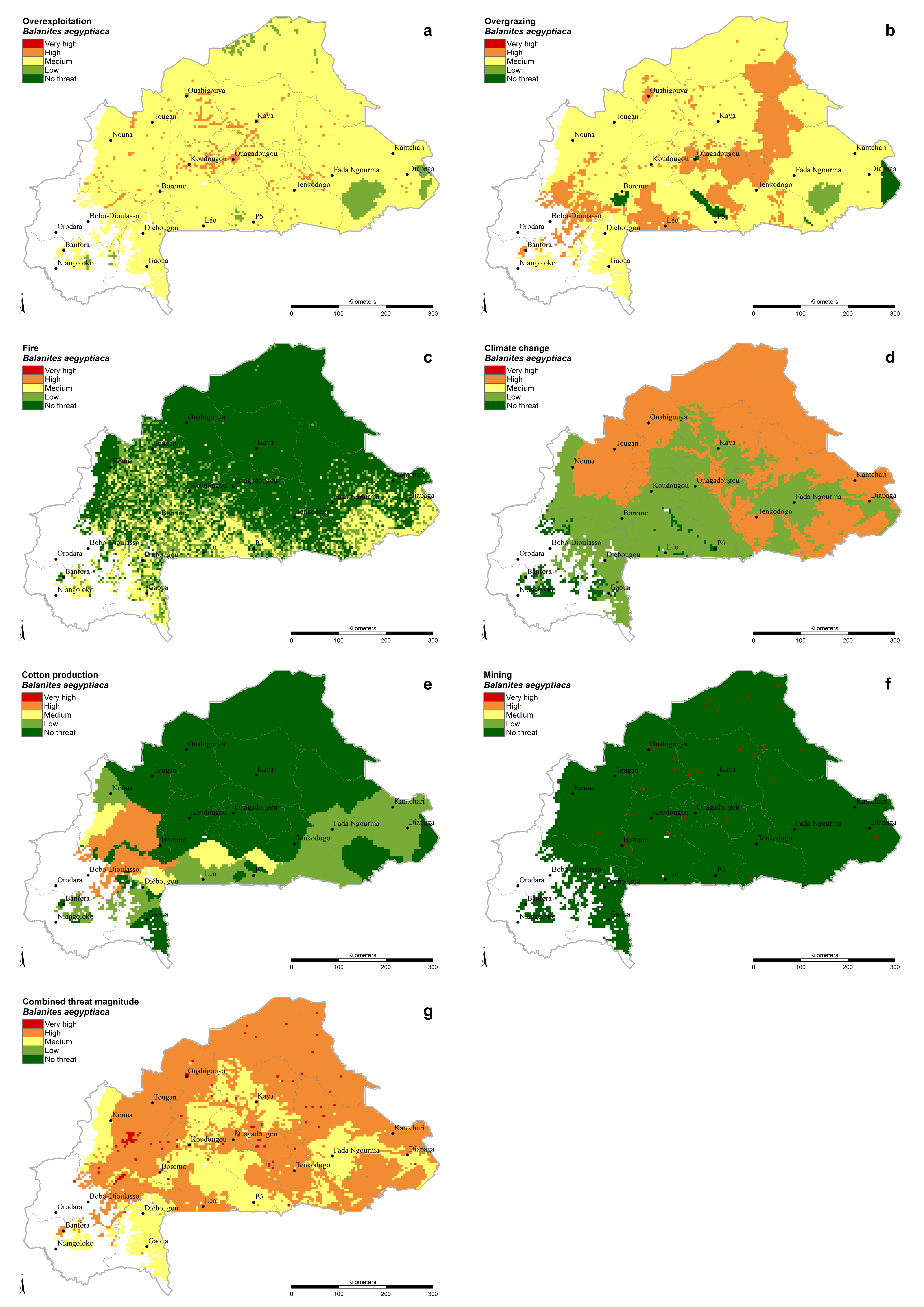

Supplement: S5 Fig — Threat magnitude levels of (A) ‘Overexploitation’, (B) ‘Overgrazing’, (C) ‘Fire’, (D) ‘Climate change’, (E) ‘Cotton production’, (F) ‘Mining’ and (G) ‘Combined threat’. (TIF) [file pone.0184457.s007.tif]

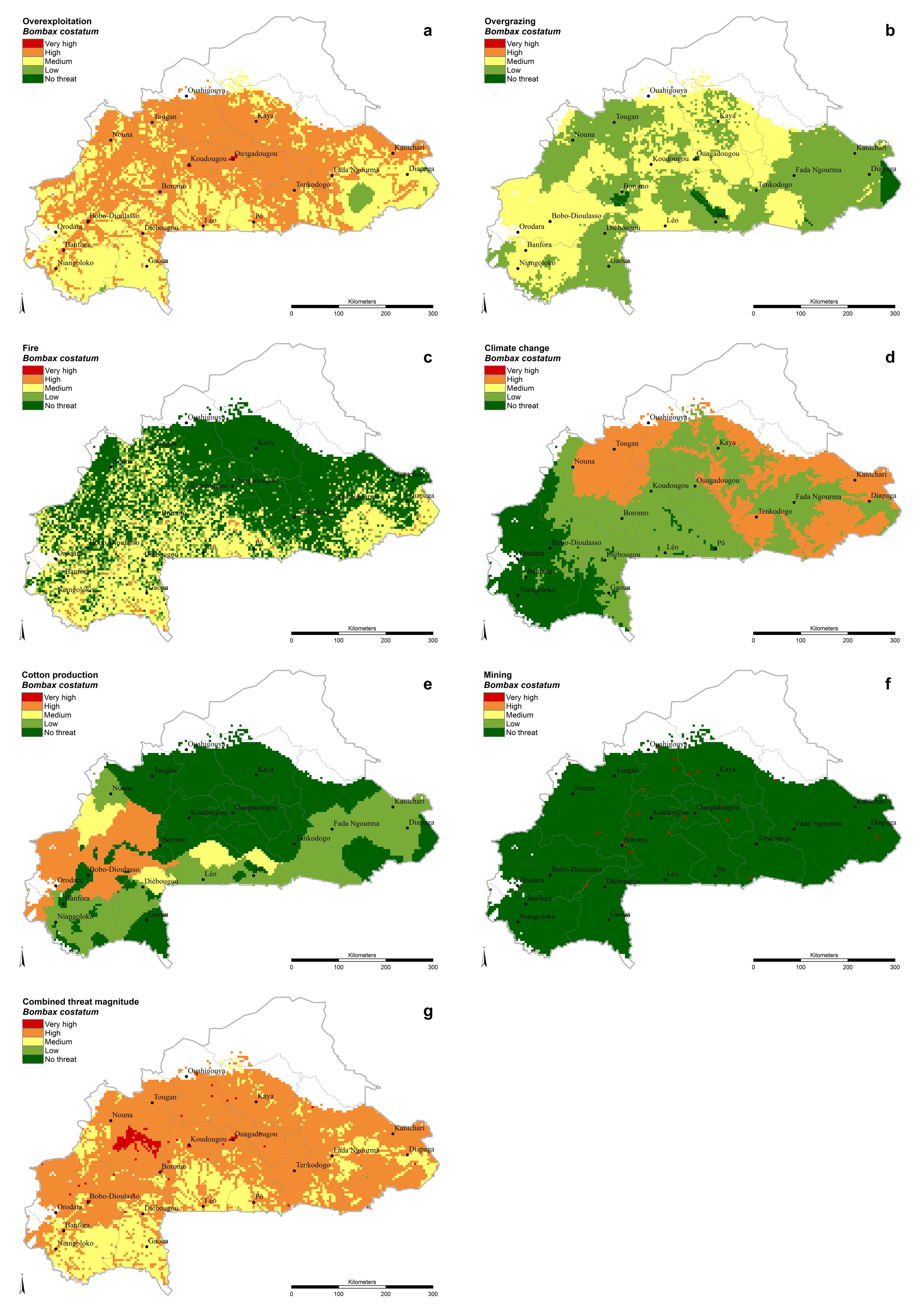

Supplement: S6 Fig — Threat magnitude levels of (A) ‘Overexploitation’, (B) ‘Overgrazing’, (C) ‘Fire’, (D) ‘Climate change’, (E) ‘Cotton production’, (F) ‘Mining’ and (G) ‘Combined threat’. (TIF) [file pone.0184457.s008.tif]

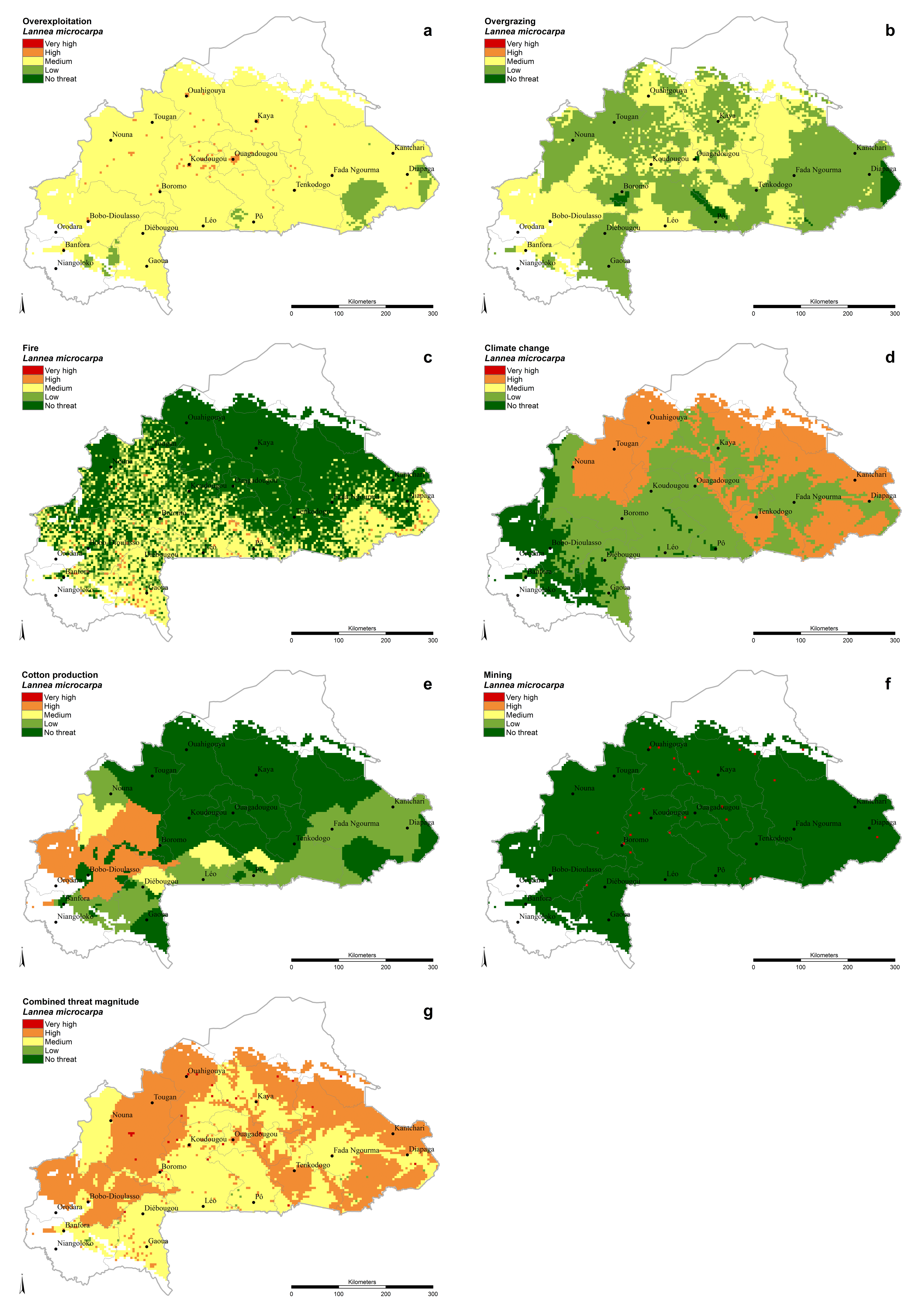

Supplement: S7 Fig — Threat magnitude levels of (A) ‘Overexploitation’, (B) ‘Overgrazing’, (C) ‘Fire’, (D) ‘Climate change’, (E) ‘Cotton production’, (F) ‘Mining’ and (G) ‘Combined threat’. (TIF) [file pone.0184457.s009.tif]

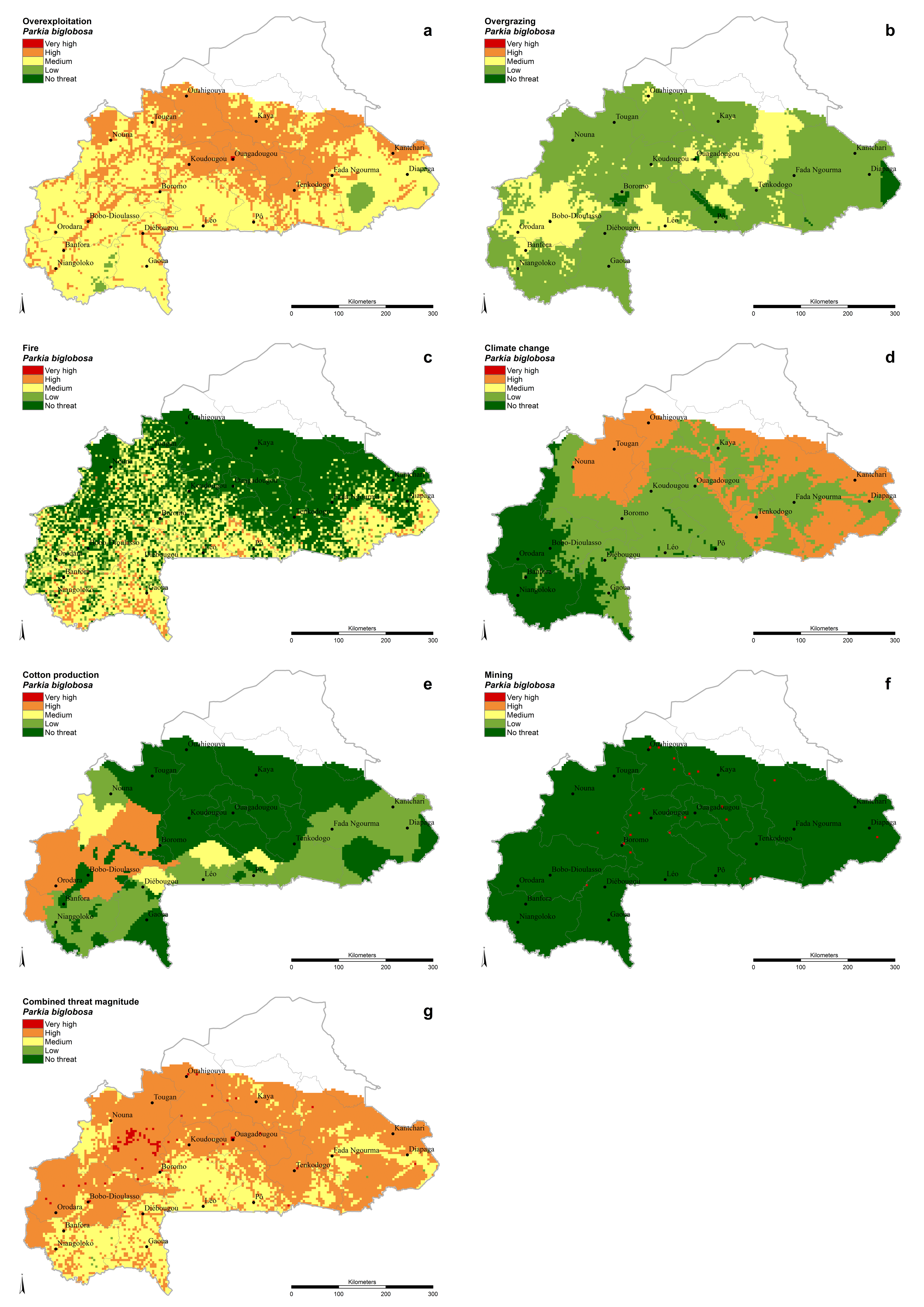

Supplement: S8 Fig — Threat magnitude levels of (A) ‘Overexploitation’, (B) ‘Overgrazing’, (C) ‘Fire’, (D) ‘Climate change’, (E) ‘Cotton production’, (F) ‘Mining’ and (G) ‘Combined threat’. (TIF) [file pone.0184457.s010.tif]

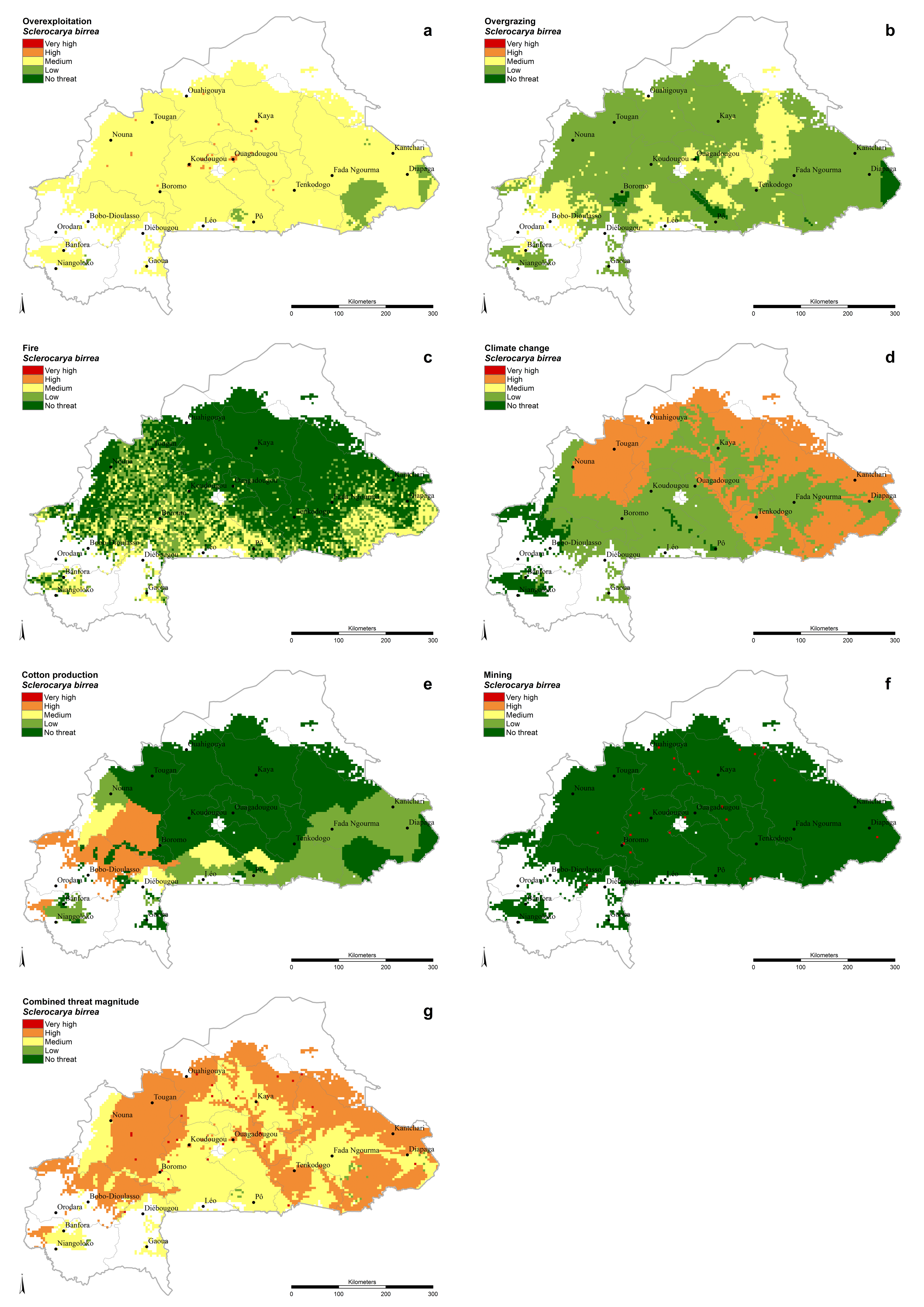

Supplement: S9 Fig — Threat magnitude levels of (A) ‘Overexploitation’, (B) ‘Overgrazing’, (C) ‘Fire’, (D) ‘Climate change’, (E) ‘Cotton production’, (F) ‘Mining’ and (G) ‘Combined threat’. (TIF) [file pone.0184457.s011.tif]

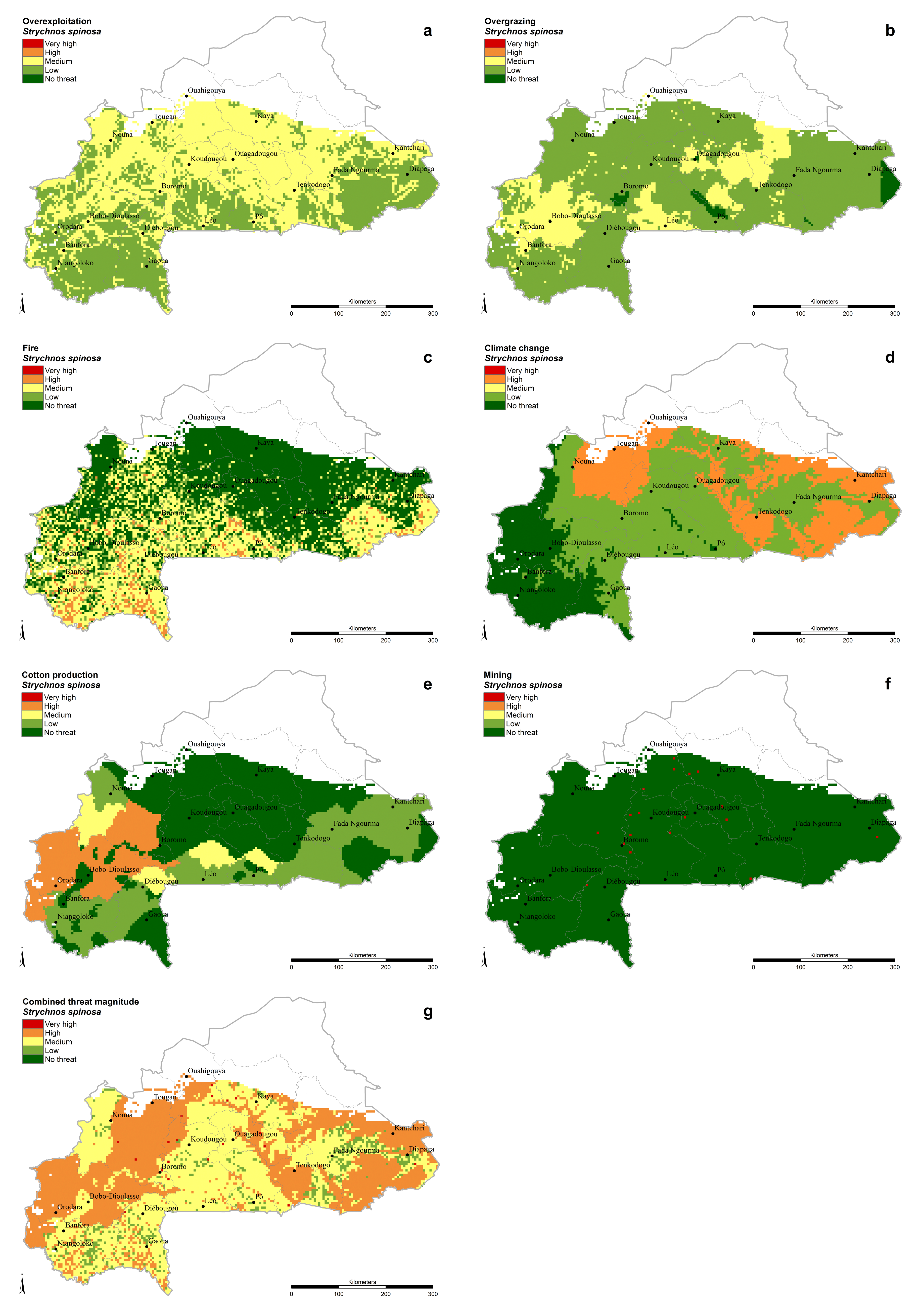

Supplement: S10 Fig — Threat magnitude levels of (A) ‘Overexploitation’, (B) ‘Overgrazing’, (C) ‘Fire’, (D) ‘Climate change’, (E) ‘Cotton production’, (F) ‘Mining’ and (G) ‘Combined threat’. (TIF) [file pone.0184457.s012.tif]

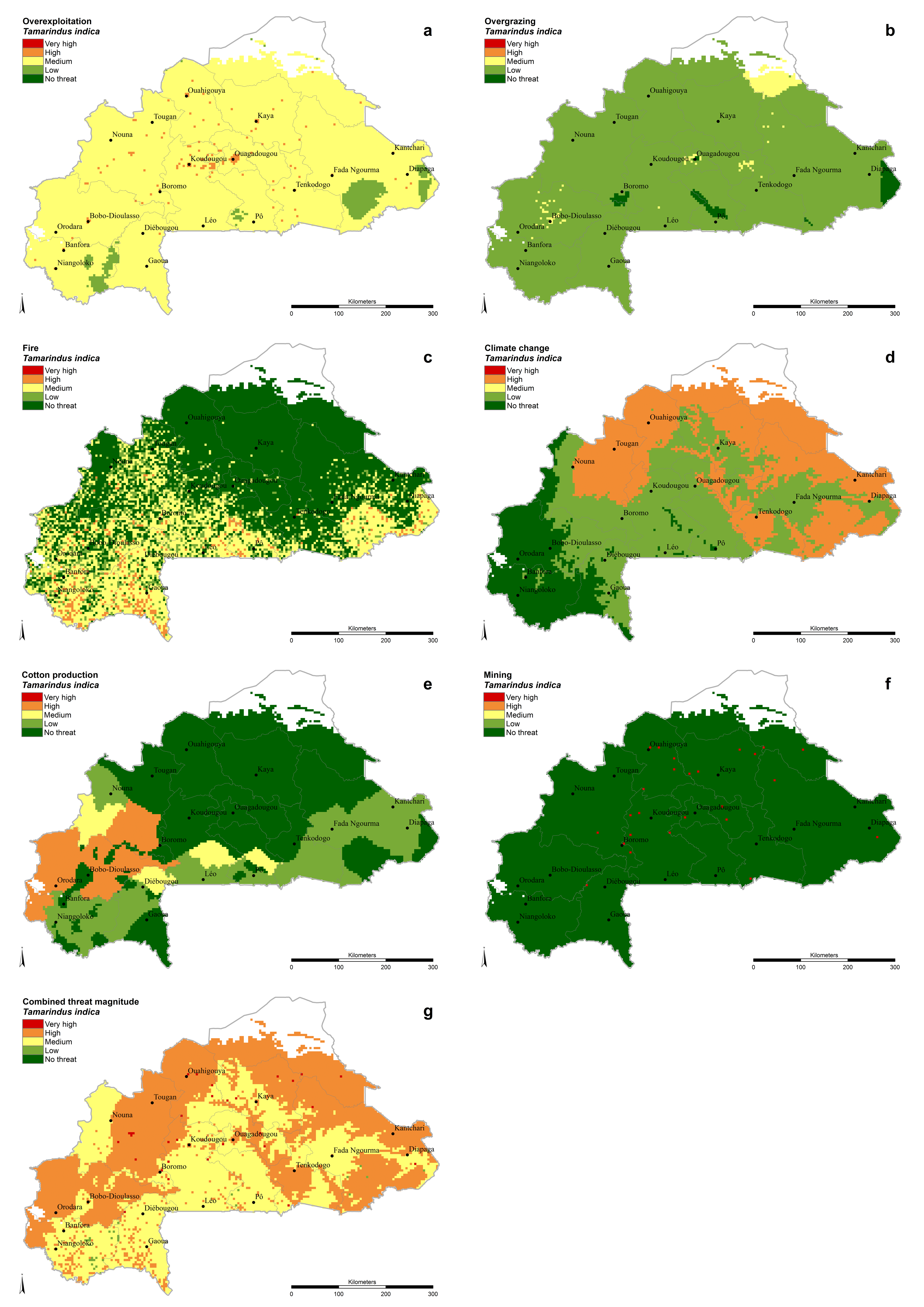

Supplement: S11 Fig — Threat magnitude levels of (A) ‘Overexploitation’, (B) ‘Overgrazing’, (C) ‘Fire’, (D) ‘Climate change’, (E) ‘Cotton production’, (F) ‘Mining’ and (G) ‘Combined threat’. (TIF) [file pone.0184457.s013.tif]

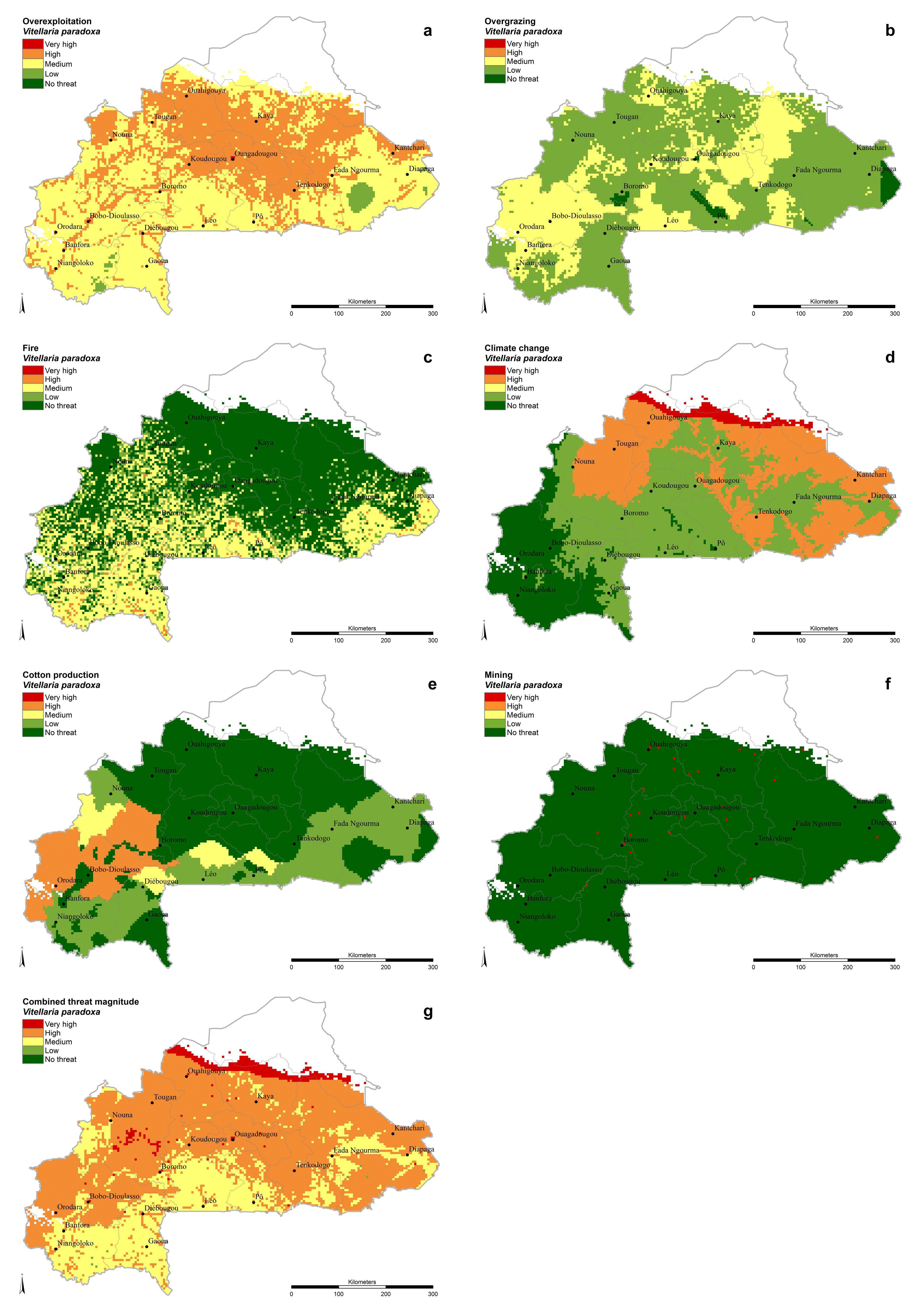

Supplement: S12 Fig — Threat magnitude levels of (A) ‘Overexploitation’, (B) ‘Overgrazing’, (C) ‘Fire’, (D) ‘Climate change’, (E) ‘Cotton production’, (F) ‘Mining’ and (G) ‘Combined threat’. (TIF) [file pone.0184457.s014.tif]

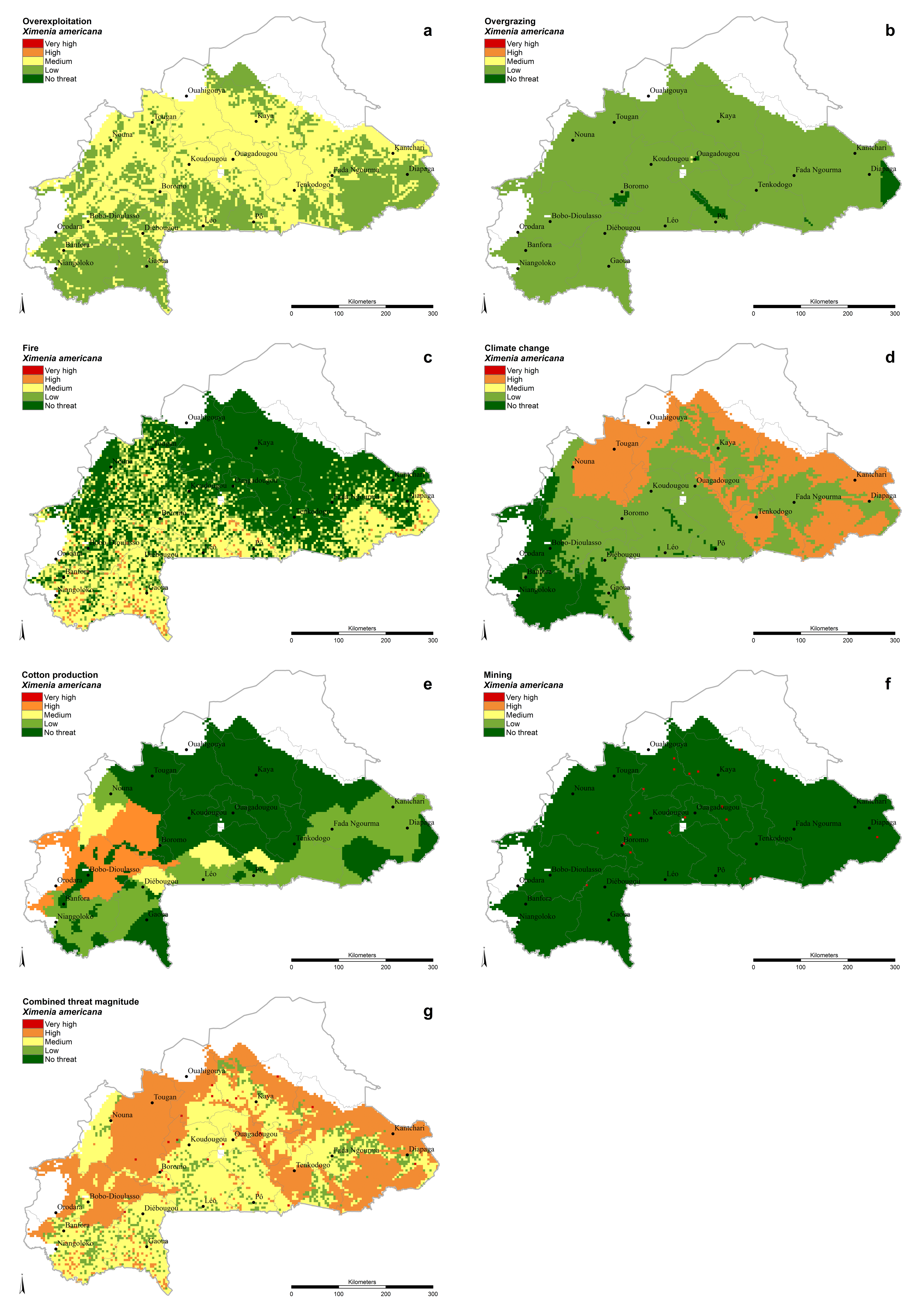

Supplement: S13 Fig — Threat magnitude levels of (A) ‘Overexploitation’, (B) ‘Overgrazing’, (C) ‘Fire’, (D) ‘Climate change’, (E) ‘Cotton production’, (F) ‘Mining’ and (G) ‘Combined threat’. (TIF) [file pone.0184457.s015.tif]

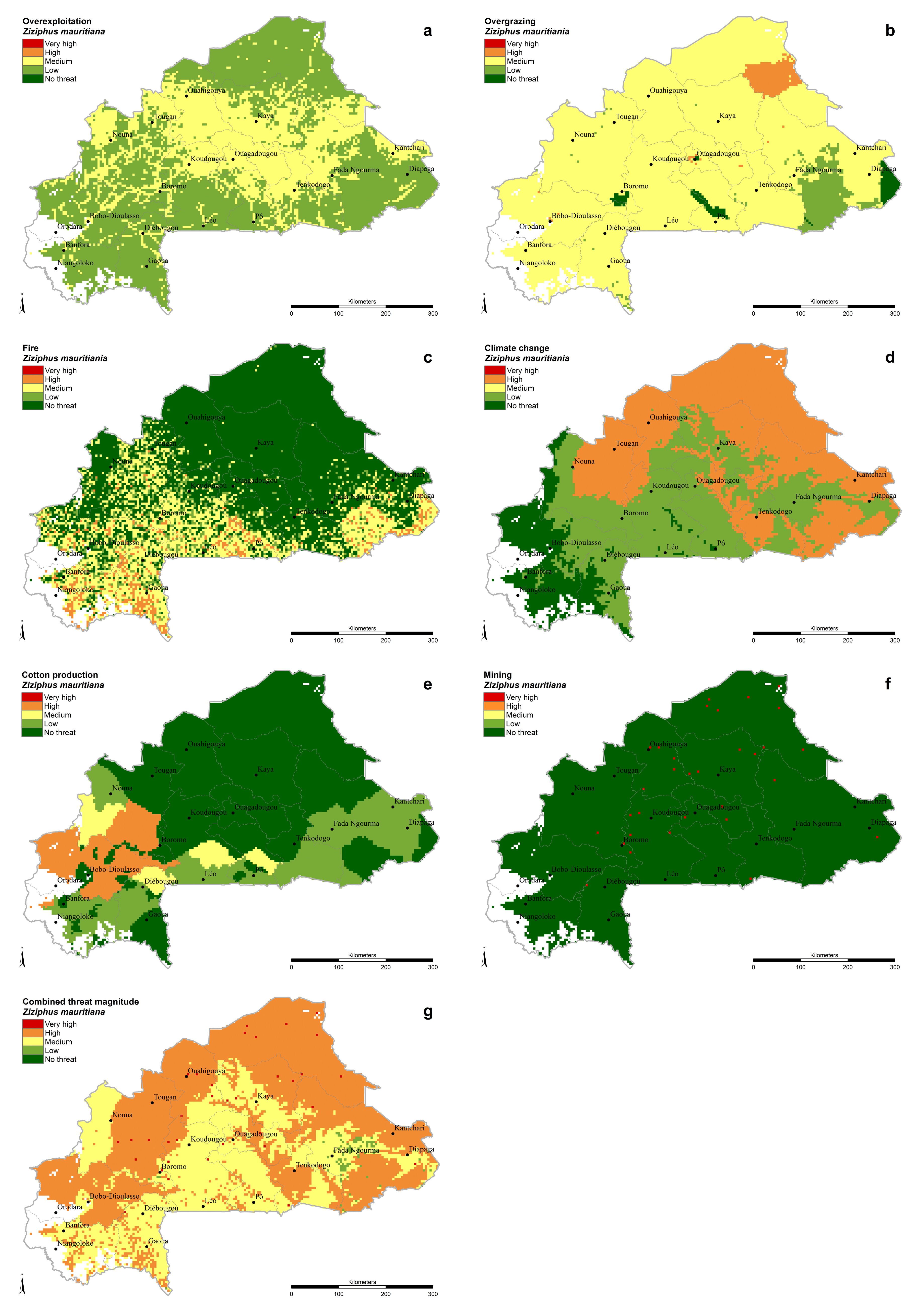

Supplement: S14 Fig — Threat magnitude levels of (A) ‘Overexploitation’, (B) ‘Overgrazing’, (C) ‘Fire’, (D) ‘Climate change’, (E) ‘Cotton production’, (F) ‘Mining’ and (G) ‘Combined threat’. (TIF) [file pone.0184457.s016.tif]
